# Bright Semiconductor Quantum Dots Shed New Light on Precision Nanomedicine for Various Diseases

**DOI:** 10.1002/smsc.202300081

**Published:** 2023-11-27

**Authors:** Peisen Zhang, Mingxia Jiao, Yilin Li, Xiaoyan Ding, Kevin J. McHugh, Lihong Jing

**Affiliations:** ^1^ CAS Key Laboratory of Colloid, Interface and Chemical Thermodynamics CAS Research/Education Center for Excellence in Molecular Sciences Center for Carbon Neutral Chemistry Institute of Chemistry Chinese Academy of Sciences Bei Yi Jie 2 Zhong Guan Cun Beijing 100190 China; ^2^ Key Laboratory of Optic-Electric Sensing and Analytical Chemistry for Life Science MOE College of Chemistry and Molecular Engineering Qingdao University of Science and Technology Qingdao 266042 China; ^3^ Department of Gastrointestinal Oncology Key Laboratory of Carcinogenesis and Translational Research (Ministry of Education) Peking University Cancer Hospital and Institute Beijing 100142 China; ^4^ Department of Cancer Center Beijing Ditan Hospital Capital Medical University Beijing 100015 China; ^5^ Department of Bioengineering Rice University Houston TX 77005 USA

**Keywords:** cancer, medical tattoo, microbial infection, semiconductor quantum dots, vascular dysfunction

## Abstract

Nanomaterials with diagnostic and therapeutic functions have exciting potential to reshape the landscape of precision medicine. Impressive progress has been made toward the design and production of innovative theranostic nanomaterials that improve disease care, motivated by their ability to simultaneously provide diagnostic information and therapeutic benefits. Herein, the state‐of‐the‐art theranostic semiconductor quantum dots (QDs) are summarized, and the diverse types of QDs designed for the diagnosis and treatment of different diseases are discussed. The opportunities and benefits of QDs are highlighted throughout using in vitro and in vivo examples aimed at addressing various clinical challenges, including cancer, vascular dysfunctions, microbial infections, and medical tattoos. Over the past several years, this area has experienced enormous growth, particularly in preclinical animal imaging and therapy, which has brought the field closer to reaching human patients. Unfortunately, several barriers to clinical translation remain. Therefore, in addition to summarizing the key results from previous in vivo studies, the lessons learned from these studies are synthesized, perspective on the future steps needed for both fundamental studies and the clinical translation of theranostic QD nanotechnology to inform future QD design is provided.

## Graphical abstract

This review provides an extensive overview of state‐of‐the‐art theranostic semiconductor quantum dots (QDs) being used for the diagnosis and treatment of different diseases. The opportunities and benefits of QD‐based nanoprobes are highlighted through the use of examples that address current unmet clinical needs. The future prospects of QD nanotechnology and the challenges facing its clinical translation are also discussed.

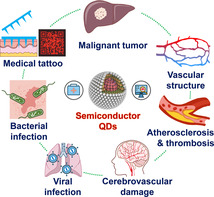

## 1 Introduction

The rapid development of nanotechnology, together with relevant detection methods and supporting facilities, has spurred a technological revolution in the biomedical field, which has led to the birth of a new discipline, known as “nanomedicine”.^[^ ^1^ ^]^ The physicochemical properties arising from nanomaterial size effects allow for the integration of more comprehensive and complicated biomedical functions in comparison with more conventional small‐molecular‐weight agents. With these unique advantages, engineered nanomaterials have played an increasingly pivotal role in the prevention, diagnosis, treatment, and prognosis of a variety of diseases.^[^ ^2^ ^]^ Nevertheless, their theranostic potential is often limited by poor spatiotemporal resolution, penetration depth, bioavailability, safety, and/or pharmacokinetics.^[^ ^3^ ^]^

Among the different nanomaterials developed thus far, quantum dots (QDs), also known as semiconductor QDs or semiconductor nanocrystals, represent an appealing theranostic nanoplatform. When the particle size of semiconductor nanocrystals is smaller than (or on the order of) their bulk exciton Bohr radii, the decreased crystal size gives rise to an increased bandgap, endowing the semiconductor nanocrystals with tunable light absorption and emission properties governed by the quantum confinement effect. Upon light irradiation, the photoexcited electron–hole pair in QDs can recombine through both radiative and nonradiative pathways. The former pathway generates fluorescence that is useful for biomedical imaging while the latter pathway produces heat useful for photothermal therapy (PTT) and photoacoustic imaging (PAI) or radicals that are useful for photodynamic therapy (PDT). Consequently, it is the unique photophysical features of semiconductor QDs endow them with the potential to both diagnose disease and act as a therapeutic. To date, QDs have been designed and synthesized with favorable fluorescent properties including a large Stokes shift, long fluorescence lifetime, narrow emission band, and a broad emission window ranging from visible to near‐infrared wavelengths, enabling researchers to tailor the optical detection strategies for a variety of clinical indications.^[^ ^4^, ^5^ ^]^ Additionally, several types of QDs have also been designed to kill harmful malignant cells or invading microorganisms, serving as good therapeutic candidates for different diseases.^[^ ^6^ ^]^ In addition to their intrinsic properties, semiconductor QDs can be also used as a carrier or a scaffold owing to the large surface–volume ratio. QD behavior and biocompatibility can generally be tuned by using the appropriate conditions in direct aqueous phase synthesis or by employing a surface modification process after organic phase synthesis. The chemistry involved in aqueous phase synthesis is very attractive due to a much richer selection of functionalities for particle surface capping agents, which enables QDs to be specifically tailored to each application. Alternatively, secondary surface modification after organic phase synthesis, which often consists of ligand exchange to replace hydrophobic ligands with hydrophilic counterparts, is another option and, fortunately, this process does not substantially impact the hydrodynamic diameter of QDs as long as the substituted ligand is small. We refer the reader to separate review articles that provide a comprehensive overview of QD surface coating strategies that alter their physicochemical and bioactive properties.[^4^, ^7^] After modifying QDs with a variety of functional ligands or biomolecules, the resultant probe can exhibit multiple capabilities, including, but not limited to, pathogen binding, catalytic activity, and selective activation.^[^ ^8^ ^]^ Together, these inherent and imparted capabilities make semiconductor QDs exciting candidates for use in theranostic applications.

Benefiting from these unique characteristics, QD‐based medical technology has been widely explored for a wide array of diseases.^[^ ^9^ ^]^ Specifically, after the enhanced penetration and retention (EPR) effect was discovered, describing the relationship between the hydrodynamic diameters of drugs and drug accumulation in solid tumors, a variety of nano‐sized semiconductor QDs have been employed to construct probes for the precise diagnosis and treatment of cancer in the past two decades.[^7^, ^10^] Through using QD‐based strategies, improved drug accumulation, ultrasensitive imaging, reduced side effects, and a number of other application‐specific benefits have been achieved. This success also led to the expanded use of QDs across a wide range of biomedical applications. For example, owing to their prolonged circulation time (with appropriate surface modification), QDs have served as imaging probes for visualizing vascular dysfunction, such as thrombosis, stroke, vascular injury, and inflammation.^[^ ^11^ ^]^ In recent years, the field's advancing knowledge of diseases and sophisticated technologies for nanoscale manipulation and microanalysis have allowed QDs to be designed to interact with complex biological systems in new ways. Researchers have been developing new QDs in an attempt to address a number of clinical challenges, such as the highly sensitive detection of pathogens,^[^ ^12^ ^]^ effective treatments for drug‐resistant microorganisms,^[^ ^13^ ^]^ and new medical recordkeeping strategies for population in low‐resource settings.^[^ ^14^ ^]^ These efforts have proven that QDs can serve as powerful tools for biomedical applications and, more importantly, can be used across a broad array of indications.

In this review, we summarize the field of diagnostic and therapeutic semiconductor QDs as a nanoplatform that is potentially applicable to many diseases. The opportunities and benefits of QD‐based nanoprobes are highlighted using examples of in vitro and in vivo studies with different disease indications, including cancer, different vascular dysfunctions, microbial infections, and medical tattoos. At last, the current challenges and future perspectives for both fundamental studies and the potential clinical translation of QD‐based biomedical agents are proposed.

## 2 Malignant Tumors

Malignant tumors are one of the leading causes of death, and the incidence rate is increasing worldwide.^[^ ^15^ ^]^ Although drug discovery and development efforts have yielded multiple fundamentally novel treatment options for cancer over the past few decades, the goal of consistent cancer eradication remains arduous due to drug resistance and the extreme complexity of cancer biology.^[^ ^16^ ^]^ At present, there are still many types of incurable cancer, thus new therapeutic approaches are urgently needed. Nano‐based diagnosis and treatment approaches have been considered promising strategies for several decades because of their potential to overcome the lack of specificity that conventional contrast agents and chemotherapeutic drugs face, which may provide clinicians with better options.^[^ ^17^ ^]^

As a category of quasi‐zero‐dimensional nanomaterials, QDs enjoy the advantages of nanoscale size and also possess their own set of unique and potentially favorable characteristics. For example, when designed with appropriate hydrodynamic diameters, QDs can readily extravasate out of the leaky vasculature of solid tumors and, more importantly, penetrate and be retained inside deep tumor tissues through the EPR effect.^[^ ^18^ ^]^ Further, with the ultra‐large specific area and rich modification sites available, different tumor‐targeting molecules can be conjugated to the QD surface, endowing them with the ability to actively target tumor cells.^[^ ^19^ ^]^ Both the improvement of tumor tissue accumulation by EPR effect and cancer cell recognition by active targeting make QDs advantageous for achieving tumor selectivity.

### 2.1 Tumor Diagnosis

Semiconductor QDs are an appealing platform for optical cancer imaging because of their tunable optical properties. More specifically, QDs can be synthesized with favorable fluorescent properties including large Stokes shifts, long fluorescence lifetimes, photobleaching resistance, and a wide variety of emission wavelengths.^[^ ^20^ ^]^ Compared with the conventional nanomaterials modified by organic small‐molecule dyes that may decompose during the complicated in vivo process, QDs with intrinsic fluorescence emission can largely ensure fluorescence remains in vivo even after multiple excitations, which may be beneficial for long‐term tumor monitoring and imaging with high sensitivity.

As early as 2004, Nie et al. have synthesized luminescent CdSe@ZnS QDs with triblock copolymer structure‐linked poly(ethylene glycol) (PEG) as biocompatible surface ligands. Through intravenous administration via the tail vein, QDs accumulated in the subcutaneous solid tumor of nude mice, presumably via the EPR effect, enabling optical tumor imaging in vivo.^[^ ^21^ ^]^ In subsequent research, they conjugated prostate‐specific membrane antigen (PSMA) antibodies to the triblock copolymers on the QD surface, endowing the QDs with the ability to actively target tumors. In the in vivo experiment, the antibody–QD conjugates realized both passive tumor targeting through the EPR effect and active tumor targeting through antibody–antigen interaction.^[^ ^22^ ^]^

The above studies demonstrated that the semiconductor QDs with satisfactory fluorescent properties can serve as an appealing nanoplatform for optical tumor imaging and diagnosis. Nevertheless, subsequent studies showed that these first‐generation Cd‐based QDs presented toxicity concerns, which largely hampered their potential for in vivo imaging. Specifically, free Cd^2+^ may be released from QDs in vivo, thereafter forming complexes with proteins that are then ingested by cells and tissues, resulting in toxicity.^[^ ^23^ ^]^ In this context, although the ZnS coating of Cd‐based QDs can prevent Cd^2+^ release to some extent in vitro, Cd^2+^ ions are still released in vivo, especially in some regions of the body where the pH is low or rich in enzymes, such as the digestive tract.

To overcome this challenge, Cd‐free QDs were developed for cancer diagnosis. These QDs were also designed to offer wide excitation and emission ranges and exhibit high fluorescence efficiency after surface passivation.^[^ ^24^ ^]^ Very recently, Jing and co‐workers successfully synthesized the Cd‐free Zn‐Cu‐In‐Se QDs with strong fluorescent emission through an aqueous synthetic route. The introduction of Zn ions can effectively restrict the nonradiative recombination pathways, thus enhancing the photoluminescence quantum yield (PL QY) of the QDs. More importantly, by adjusting the molar ratio of Zn and Cu, the PL emission maxima of QDs can be tuned from 630 to 800 nm, while the diameter of the QDs remained nearly unchanged, demonstrating the particle size‐independent manipulation of PL properties. Leveraging this relationship, a series of QDs with the same size but different emission wavelengths were used to construct multiple targeted nanoprobes for multiplexed mapping of biomarkers distribution within a single tumor cell with high spatial resolution in vitro.^[^ ^25^ ^]^

Compared to visible light, near‐infrared (NIR) light—particularly those falling in the second near‐infrared window (NIR‐II, between ≈ 1000 and 1700 nm)—has several key advantages for medical imaging applications.^[^ ^26^ ^]^ Due to the low autofluorescence and light absorption/scattering of biological tissues in this wavelength range, the tissue penetration depth and spatial resolution of NIR‐II optical imaging are greatly improved.^[^ ^27^ ^]^ More importantly, with the development of the InGaAs detectors, NIR‐II light can be collected with high efficiency. Under this circumstance, a substantial amount of effort has been dedicated to developing NIR‐II imaging probes with small‐molecular organic dyes, rare‐earth‐based nanoparticles, aggregation‐induced emission luminogens, and semiconductor QDs. Specifically, the introduction, merits, and demerits of these NIR‐II fluorophores have been comprehensively discussed, which can be found in the recent review articles.^[^ ^28^ ^]^ Among these candidates, semiconductor QDs formed by narrow bandgap materials have been explored for their tunable NIR‐II emission derived from the quantum confinement effect.^[^ ^29^ ^]^ In 2012, Dai and co‐workers reported the use of Ag_2_S QDs with high PL QY (15.5 %) NIR‐II emission at approximately 1200 nm while performing optical imaging of tumor xenografts. The strong fluorescent intensity of QDs allowed for deep inner organ registration, dynamic tumor contrast, and fast tumor detection in vivo with a high signal‐to‐background ratio.^[^ ^30^ ^]^ In the subsequent studies, Wang and co‐workers presented an Ag_2_S QD‐based nanoprobe combining two imaging modalities for preoperative diagnosis and image‐guided brain tumor resection. The PEGylated Ag_2_S QDs with intrinsic NIR emission were modified with Gd‐DOTA on the surface to impart MRI contrast. After intravenous injection, these Gd‐Ag_2_S nanoprobes accumulated in the U87MG glioma tumor, which enabled the precise evaluation of tumor morphology in *T*_1_‐weighted imaging. In addition, through the intraoperative guidance of NIR‐II fluorescence imaging, the brain tumor can be precisely excised with far fewer residual tumor cells remaining at the surgical site after surgery.^[^ ^31^ ^]^ Very recently, Gao and co‐workers developed Ag_2_S QD‐based self‐illuminating and NIR‐II emissive nanoprobes to enhance the penetration depth and spatiotemporal resolution of conventional bioluminescence imaging. To do this, luciferase enzyme molecules were attached to the surface of QDs. Owing to the bioluminescence resonance energy transfer (BRET) effect between the luciferase enzyme and Ag_2_S QDs in the presence of substrate, the emission range could be extended into the NIR‐II region as Ag_2_S QDs served as energy acceptors. This bioluminescence/fluorescence dual‐modality nanoprobe was applied to in vivo tumor imaging in mice and showed that NIR‐II bioluminescence exhibited an approximately twofold higher signal‐to‐noise ratio in comparison with those obtained under fluorescence mode.^[^ ^32^ ^]^

**Figure 1:**
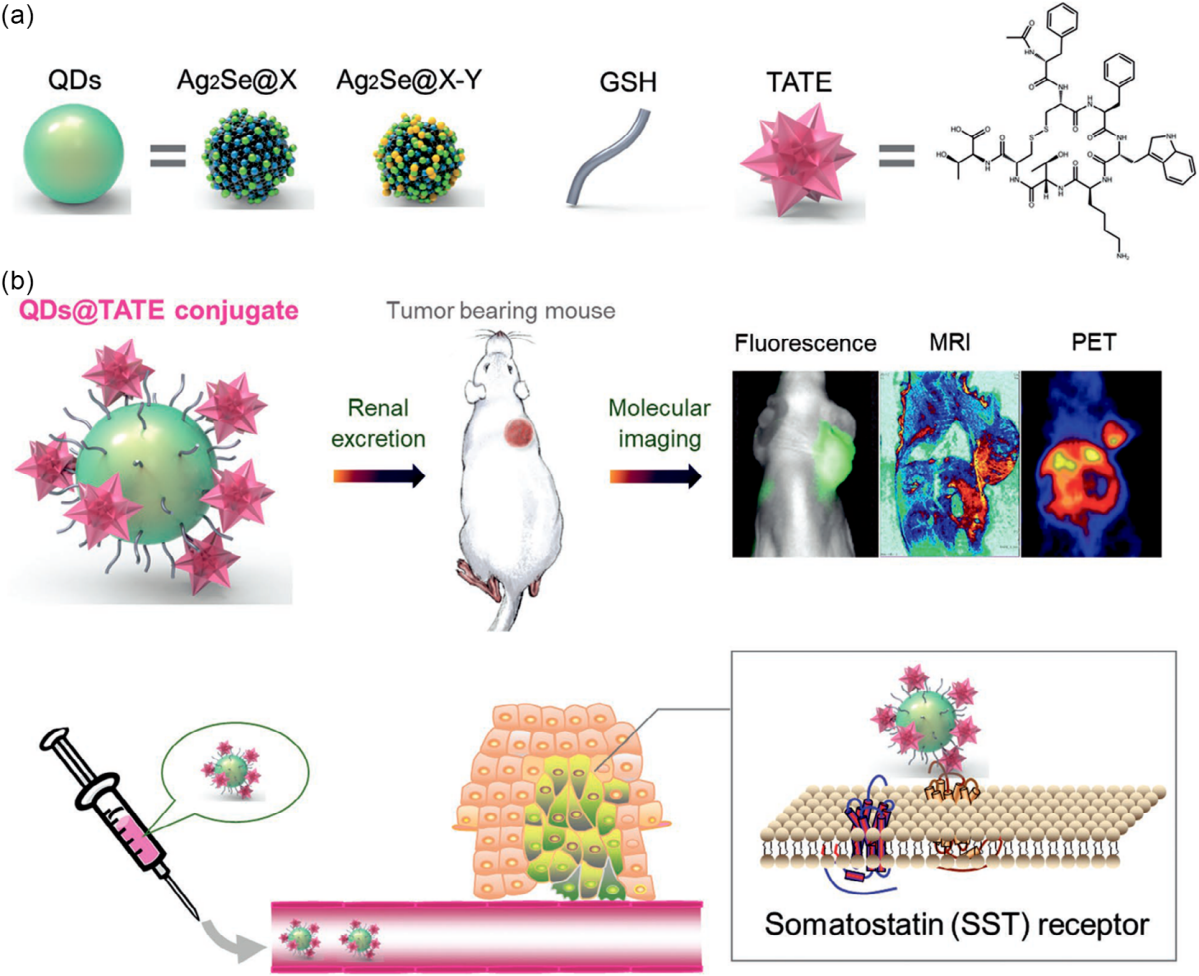
a) Ultrasmall Ag_2_Se QDs were covered by an active oxygen layer after reaction with NaOH, allowing for facile incorporation of both active and inert metal ions (e.g., Ag_2_Se@X could be Ag_2_Se@Mn, Ag_2_Se@Cd, Ag_2_Se@Hg, Ag_2_Se@Cu as dual‐modal probes, and Ag_2_Se@X‐Y could be Ag_2_Se@Mn‐Cu and Ag_2_Se@Mn‐Y as trimodal probes). b) Multifunctional QDs@peptide conjugate was achieved by coupling octreotate peptide onto the surface of QDs. Specifically, Ag_2_Se@Mn‐^64^Cu QDs were used for NIR fluorescence, MRI, and PET imaging. Reproduced with permission.^[^ ^35^ ^]^ Copyright 2019, Wiley‐VCH.

Although NIR light‐based imaging technology holds great promise, the intrinsic limitations (spatial resolution and tissue penetration depth) associated with optical imaging still prevent the precise visualization of deep anatomical structures and pathological features. To address this issue, magnetic resonance imaging (MRI), an imaging modality that can provide high‐resolution tomographical images with unlimited penetration depth, has been employed to complement optical imaging. Following this approach, Jing et al. synthesized core–shell CdTe@ZnS QDs through an aqueous synthetic route in which paramagnetic Mn^2+^ ions were doped inside a thin ZnS shell. These QDs not only exhibited strong fluorescence with high PL QY (up to 45%) due to the effective surface passivation but also displayed high ionic longitudinal molar relaxivity, *r*_1_, at up to 10.7 mM^−1^ s^−1^ under a 3.0 T magnetic field, endowing them with promising optical/MR imaging performance.^[^ ^33^ ^]^ On this basis, they further developed Cd‐free optical/MR QD imaging probes for dual‐modality tumor diagnosis. In this work, Cd‐free CuInS_2_@ZnS:Mn QDs, in which the Mn^2+^ ions were mainly located in the ZnS shell to effectively balance the optical and magnetic properties, were designed and synthesized. In vitro experiments revealed that the IC_50_ of Cd‐free CuInS_2_@ZnS:Mn QDs on HeLa cells was more than 7000‐fold higher than that of thioglycolic acid‐stabilized CdTe QDs, highlighting the greatly reduced toxicity of these Cd‐free QDs. In in vivo imaging experiments, not only could subcutaneous tumor xenografts can be clearly visualized in BALB/c nude mice through optical imaging, but deeper intraperitoneal tumor xenografts could be visualized by MRI.^[^ ^34^ ^]^ In addition to optical imaging and MRI, nuclear imaging techniques, including positron emission tomography (PET) and single photon emission computed tomography (SPECT), have been widely used for noninvasive in vivo imaging due to their high sensitivity. Chen et al. synthesized a NIR fluorescence, MRI, and PET nanoprobe compatible with trimodal imaging through a chelator‐free and post‐synthesis metal attachment strategy. The ultrasmall Ag_2_Se QDs can attach both active/inert metal ions through the layer of active oxygen on the surface, thereby enabling the tri‐modality imaging. After modification with a targeting octreotate peptide (TATE), the resulting nanoprobe achieved a satisfactory tumor‐to‐tissue ratio in somatostatin receptor subtype‐2‐positive solid tumors. In addition, after tumor imaging, the nanoprobes were eliminated in the urine, which highlighted their favorable safety profile (**Figure** **1**).^[^ ^35^ ^]^

In addition to the direct diagnosis of solid tumors, QDs can be also employed to evaluate the efficacy during the antitumor treatment process. For example, the temporal variation of tumor progression‐associated biomarkers in the local microenvironment and immune organs can be monitored by QD‐based nanoprobes, thereby providing more information about the prognosis and outcomes. Zhong et al. showed the combined heterogeneous bio‐distributions of PD‐L1 and CD8^+^ T cells in the tumor‐bearing mice in vivo through a combination of erbium‐based nanoparticles (ErNPs) and PbS QDs. Although these two kinds of nanoparticles exhibited NIR‐II emission at around ≈1600 nm, their different luminescence lifetimes allowed their signals to be easily distinguished from one another during imaging. In the subsequent two‐plex molecular imaging study with anti‐PD‐L1 mAb‐labeled ErNPs and anti‐CD8α mAb‐labeled PbS QDs, the distribution of PD‐L1 and CD8^+^ T cells was simultaneously visualized in NIR‐II window in vivo. By analyzing the tumor PD‐L1 expression level and immune cell status, the potential efficacy and outcomes of cancer immunotherapy could be evaluated and predicted.^[^ ^36^ ^]^ Apart from monitoring tumor progression‐associated biomarkers, QDs can also be used for tracking vaccine dynamics and realizing the visualization of vaccine fate in vivo. In Liu's study, different types of core–shell semiconductor QDs with various emission wavelengths were conjugated with antigen (ovalbumin, OVA) and adjuvant (unmethylated cytosine‐phosphate‐guanine, CpG) and adopted as trackers and nanocarriers. Benefiting from the strong brightness and excellent photobleaching resistance of QDs, the long‐term spatiotemporal tracking of the nano‐vaccine in vivo can be realized, which could help guide the vaccination interval of the nano‐vaccine.^[^ ^37^ ^]^

### 2.2 Tumor Treatment

In addition to tumor diagnosis, semiconductor QDs also present remarkable characteristics suitable for tumor therapy. The aforementioned passive and active targeting abilities of QDs can improve cancer treatment precision and the unique physicochemical properties also endow QDs with antitumor features.^[^ ^38^ ^]^ Specifically, the intrinsic defects, such as vacancies, interstitial defects, and antisite defects, in some categories of semiconductor QDs may result in complicated nonradiative relaxation pathways of photoexcited charge carriers.^[^ ^39^ ^]^ Although these defect‐induced nonradiative transitions may lead to energy loss, hampering the fluorescence efficiency of QDs, the process can generate heat and/or radicals under light irradiation.^[^ ^40^ ^]^ Therefore, apart from the radiative recombination of QDs that generates fluorescence, the nonradiative recombination process can be used to promote heating and/or radical production for tumor phototherapy to make maximum use of the absorbed photon energy. Although a large number of multifunctional nanoprobes that can offer both imaging and photothermal/photodynamic capabilities have been reported, most of these nanoprobes have been obtained by combining multiple functional components into a single nanoplatform. Unfortunately, these probes may be unstable upon metabolism in vivo and even dissociate during the circulation in bloodstream before reaching the lesion, making it difficult to achieve their multifunction and acquire accurate diagnosis information.^[^ ^41^ ^]^ In comparison, with the unique intrinsic photophysical properties, semiconductor QDs can enable both optical diagnosis and photothermal/PDT or even be useful for real‐time image‐guided cancer therapy without the need for additional functional moieties. Based on this principle, Qu and co‐workers reported the facile synthesis of 2–3 nm boron QDs which exhibited not only the ability to strongly absorb NIR light but also enhanced nonradiative transitions due to the abundance of defects at the exposed edges derived from the empty orbit of boron atoms. This nonradiative process endowed QDs with high photothermal conversion efficiency (PCE) of 57% under 808 nm laser excitation. In in vivo experiments, these boron QDs were able to successfully diagnose a subcutaneous tumor in mice through PAI and exhibited satisfactory PTT to eradicate the tumor cells in vivo.^[^ ^42^ ^]^ In another study, Leong et al. synthesized a series of biocompatible CoS_*x*_ QDs with variable degrees of defects. Interestingly, they found the PCE value and Fenton‐like activity can be balanced via defect levels, i.e., with increasing defect sites, the Fenton‐like activity will be improved to produce more hydroxyl radicals while PCE declines correspondingly. Using this property, CoS_*x*_ QDs were successfully optimized to provide both satisfactory PDT and PTT performance. In addition, under 808 nm light irradiation, the enhanced local temperature would further improve the catalytic rate to promote ROS generation. Therefore, CoS_*x*_ QDs successfully eliminated the primary subcutaneous solid tumor of mice through the synergistic effect of PDT and PTT while preventing lung metastasis.^[^ ^43^ ^]^

**Figure 2:**
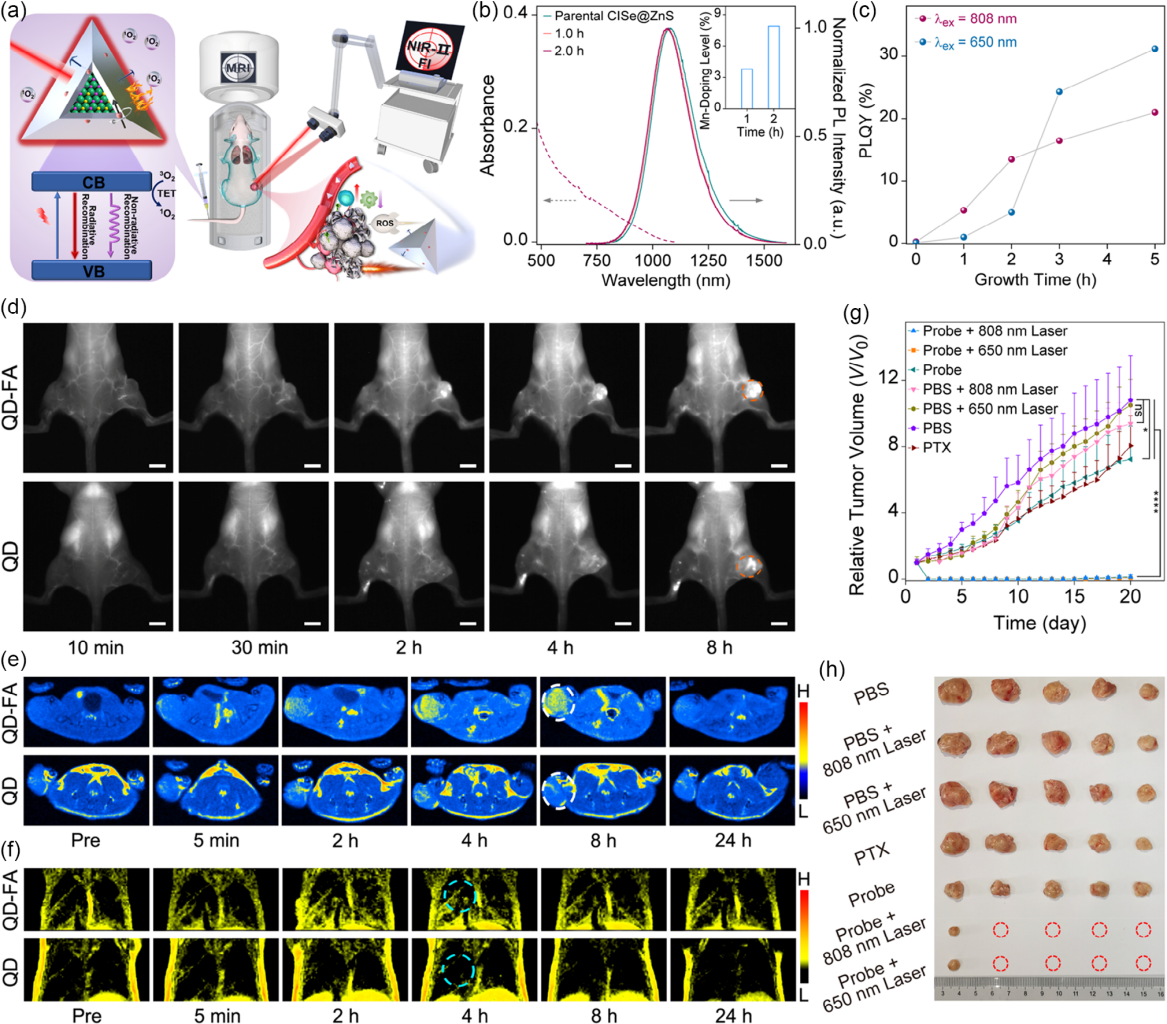
a) Scheme of CISe@ZnS:Mn QDs with built‐in multifunctionality. b) PL spectra of CISe@ZnS:Mn QDs with different Mn doping times. The dashed line depicts absorption by the sample doped for 2 h. c) PL QYs of QDs during 3 h of ZnS shell growth and subsequent 2 h of Mn doping. d) In vivo NIR‐II fluorescence imaging of subcutaneous tumors in mice, e) MR imaging of subcutaneous tumors, and f) lung metastasis of mice recorded at different time points after the intravenous injection of folic acid‐modified QDs (QD‐FA, probe) and mother QD. Scale bars: 5 mm. g) Relative tumor volumes after the phototherapy with the probe and other treatments. h) Representative images of 4T1 tumor tissues from each group harvested 20 days posttreatment. Reproduced with permission.^[^ ^44^ ^]^ Copyright 2022, American Chemical Society.

Very recently, Jing and co‐workers designed and synthesized multifunctional Mn^2+^‐doped CuInSe_2_@ZnS (CISe@ZnS:Mn) QDs that can not only achieve the accurate localization of tiny metastases but also act as potent tumor ablation agents (**Figure** **2**a). By leveraging the optimal growth kinetics of a ZnS shell and Mn doping levels to promote radiative recombination pathways and boost NIR‐II fluorescence, the CISe@ZnS:Mn QDs exhibited high NIR‐II window fluorescence with absolute PL QY up to 31.2% (Figure 2b **,**c). After modifying the surface of these QDs with folic acid (FA), the resulting tumor targeting nanoprobe (QD‐FA) was able to specifically enhance the signal from subcutaneous solid tumors via NIR‐II optical imaging and MRI without clinically relevant limitations in imaging depth, conferring the ability of this QD‐FA probe to image small metastases in the lungs (Figure 2d **–**f). Taken together, these strongly NIR‐II emissive and magnetic QDs possess promising potential for monitoring the temporal evolution of tumor progression while sensitively detecting possible metastasis, which is critical for preventing spread during recurrence. In addition to the diagnostic benefits, the nonradiative relaxation pathways of the QDs were able to effectively convert photon energy to both heat and radicals under NIR light irradiation, which was successfully employed for PTT and PDT. Through histological analysis, these QD‐based tumor phototherapies also initiated an anticancer host immune response, which complemented the therapeutic efficacy of phototherapy (Figure 2g,h). This work strongly highlights the built‐in multifunctionality of QDs applicable to sensitive dual‐modality imaging and intensive combination therapies.^[^ ^44^ ^]^

Apart from the aforementioned QDs with nonradiative, relaxation‐induced photothermal effects, black phosphorus (BP) QDs are another emerging type of nanomaterial with potential in the biomedical field.^[^ ^45^ ^]^ As a semiconductor material with puckered honeycomb structure, BP exhibits a thickness‐dependent direct bandgap, ranging from 0.3 eV for bulk to 2.0 eV. On this basis, the broad absorption of BP QDs can be realized by regulating the layer number. This widely tunable bandgap of BP QDs allows for the broad absorption from visible to NIR wavelengths, providing substantial advantages for PA imaging and PTT.^[^ ^46^ ^]^ Additionally, under laser irradiation, the energy from the excited excitons of BP QDs can be transferred to the nearby ground‐state ^3^O_2_, promoting the generation of reactive ^1^O_2_ species, thereby initiating the PDT process.^[^ ^47^ ^]^

In 2015, Chu et al. synthesized BP QDs with a lateral size of ≈2.6 nm and a thickness of ≈1.5 nm using a liquid exfoliation approach. These QDs possessed a large extinction coefficient of 14.8 Lg^−1^ cm^−1^ at 808 nm and exhibited a photothermal conversion efficiency of 28.4%. With this satisfactory photothermal performance, these QDs could be adopted as photothermal agents to effectively ablate cancer cells in vitro after PEG modification.^[^ ^48^ ^]^ In the subsequent studies, by using a similar synthesis route, Guo et al. prepared PEGylated BP QDs for combined PTT and PDT therapy. These biocompatible QDs exhibited strong NIR absorbance, enabling photothermal conversion, and can generate cytotoxic ^1^O_2_ upon the 808 nm laser irradiation. Through the synergistic effects of PTT and PDT, the intratumoral administration of QDs was able to successfully inhibit subcutaneous tumors in mice.^[^ ^49^ ^]^

Apart from PEG modification, coating QDs with cell membranes has also been employed to enhance the biocompatibility and bioavailability of BP QDs. For example, Liang et al. developed biomimetic erythrocyte membrane‐camouflaged BP QDs, which possessed both NIR photothermal performance and photothermal‐enhanced GOx‐like activity. After intravenous administration, these BP QD‐based nanomaterials accumulated in the solid tumor due to the presence of the homing peptide iRGD inserted into the outer cell membrane. Under 808 nm laser irradiation, these nanomaterials quickly enhanced the local temperature of tumors while boosting GOx‐like activity to generate more cytotoxic ROS and synergistically kill cancer cells. In vivo tumor treatment revealed that these BP QD‐based nanomaterials successfully inhibited tumor growth, as hypothesized.^[^ ^45^ ^]^ Very recently, Xiao et al. packaged BP QDs into exosome vectors (EXO) derived from tumor cells through electroporation. The coating of EXO cell membranes enhanced the stability of inner BP QDs and improved the underlying nanomaterial's biocompatibility, stealth nature (i.e., immune evasion), and tumor penetration. After intravenous administration, these nanomaterials actively accumulate within the solid tumors through EPR and homing effects. In vivo experiments indicated that upon NIR light irradiation, the local temperature of the solid tumors could be significantly enhanced with the help of BP QDs, which damaged tumor cells and inhibited their proliferation.^[^ ^50^ ^]^

**Figure 3:**
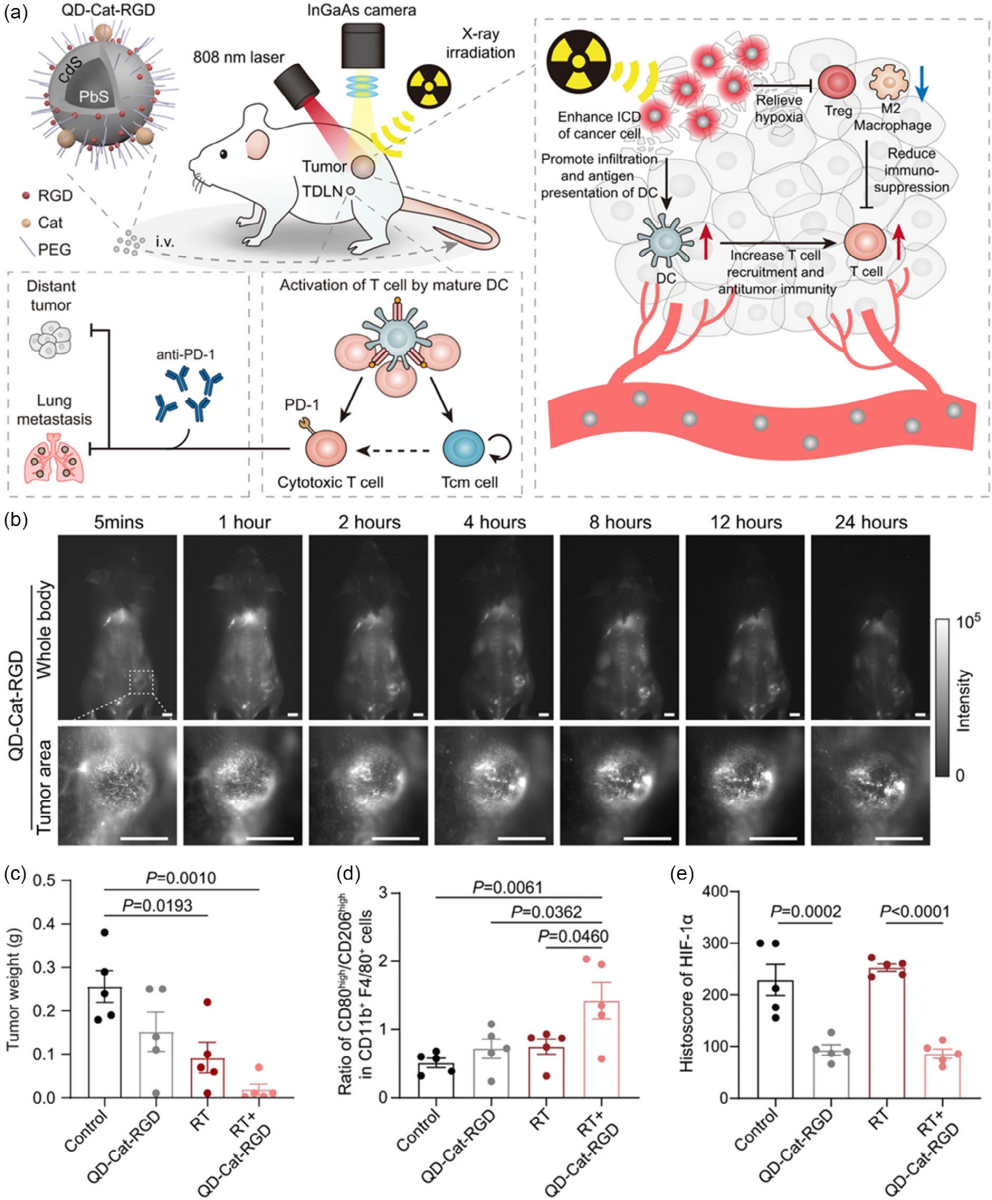
a) A scheme of QD‐Cat‐RGD nanoprobe‐based RT that boosts the antitumor immune response and abscopal effect to inhibit cancer metastases. b) Whole‐body and high‐magnification fluorescent images of 4T1 tumor‐bearing mice intravenously injected with QD‐Cat‐RGD nanoprobes and imaged in the NIR‐II window. Scale bars: 5 mm. c) Histogram of 4T1 tumor weight. d) The ratio of M1/M2 macrophages in tumor microenvironment after treatment. e) Quantitative analysis of HIF‐1α immunostaining. Reproduced with permission.^[^ ^51^ ^]^ Copyright 2021, Nature Publishing Group.

In addition to PDT and PTT effects, several QDs with high atomic number (high‐Z) elements, such as Pb or Ag, which can be served as radiosensitizers to promote radiation energy deposition to enhance cancer radiotherapy (RT), have been developed. Sun et al. constructed a stabilized theranostic nanoprobe based on NIR‐II‐emitting QDs, to accurately diagnose solid tumors with high spatial resolution, boost the radiosensitivity and immunogenicity of cancer cells, and relieve tumor hypoxia to enhance the sensitivity to RT. After capping the PbS@CdS QDs with catalase (Cat), arginine–glycine–aspartate (RGD) peptides, and PEG ligands, the resulting probes actively accumulated in the solid tumors, enabling the cancer diagnosis while guiding precision radiotherapy. Importantly, with the high‐Z element Pb, these probes exhibited good performance as a radiosensitizer, which not only promoted the efficacy of RT but also amplified immunogenic cancer cell death. Additionally, through the decomposition of H_2_O_2_ by Cat on the probes, O_2_ can be generated to enhance the radio sensitization of cancer cells and relieve the immunosuppressive status of the hypoxic tumor microenvironment. This QDs‐based strategy may have the potential to enhance both the therapeutic precision and efficacy of RT in tumors (**Figure** **3**).^[^ ^51^ ^]^

## 3 Vascular Dysfunctions

Vascular dysfunctions, primarily the abnormality of vascular anatomical structures, hemodynamic alterations, and up‐ or downregulation of molecular expression, are closely related to the onset of various diseases, such as stroke, thrombosis, vascular inflammation, and aneurysm. The accurate and early detection of vascular dysfunction is of great clinical significance because it allows for the optimization of the treatment plan, thereby improving the prognosis and decreasing the adverse effects.^[^ ^52^ ^]^

In general, the detection strategies used for vascular diseases can mainly be divided into two types of strategies. One is the detection of vascular anatomical structures. Many vascular diseases are accompanied by abnormal variations of vascular anatomy, such as microvascular proliferation, vascular swelling, or stenosis. For example, atherosclerotic plaques often lead to vascular stenosis and even blockage. In some chronic ischemic diseases, small collateral vessels can be formed to provide a supplemental blood supply for the ischemic tissues.^[^ ^53^ ^]^ Through medical imaging, these anatomical changes can be readily visualized.^[^ ^54^ ^]^ The other strategy used relies on the detection of pathological molecules. Vascular endothelial cells lining the luminal surface of the vasculature play key regulatory functions in the body; however, their surface molecular expression can be altered under pathological conditions. Accordingly, the abnormal expression of these pathological molecules can be adopted as the specific targets of vascular diseases. For example, several integrins, selectins, and cell adhesion molecules on the membrane of endothelial cells are upregulated when inflamed, which can serve as the target molecules for detecting inflammation.^[^ ^55^ ^]^ Considering that changes in vascular anatomy are usually attributed to the cumulative effect of aberrant molecular events, molecular changes typically precede anatomical defects; therefore, detecting pathological molecules detection may enable earlier vascular disease diagnosis.

Interestingly, semiconductor QDs exhibit remarkable characteristics suitable for the diagnosis of vascular diseases. On the one hand, owing to their outstanding fluorescence properties, the anatomical structures of the vasculature can be readily mapped through optical imaging. On the other hand, the various surface modification strategies allow for the construction of QD‐based targeting probes for pathological molecule detection. Therefore, it is possible that through the QD‐based imaging strategy, both the anatomical abnormalities of micro‐blood vessels and pathological molecular changes of vascular cells can be used together for diagnosis, which will almost certainly enhance diagnostic accuracy and provide more information for clinicians.

### 3.1 Imaging of Vascular Anatomy

High‐resolution angiography can provide anatomical information on vascular abnormalities, which is very important for the early diagnosis of many vascular diseases. In clinical practice, different angiography modalities, including digital subtraction angiography (DSA), computed tomography angiography (CTA), and magnetic resonance angiography (MRA), have been widely used in revealing the anatomical structures of the vasculature.^[^ ^56^ ^]^ In the past few years, optical imaging technologies, offering noninvasiveness, high sensitivity, and real‐time feedback, have been widely applied in vascular mapping visualization and perfusion imaging.^[^ ^57^ ^]^ In comparison with the visible and NIR I (400–900 nm) imaging windows, which are compromised by the absorption, scattering, and autofluorescence from tissues, the NIR‐II imaging window (1000–1700 nm) possesses improved spatial resolution and a higher signal‐to‐background ratio, thus showing more promise for angiography of vascular anatomy.^[^ ^58^ ^]^ A variety of semiconductor QDs with bright NIR‐II fluorescence and the potential for the noninvasive imaging of anatomical structures of vessels have been synthesized to date.

**Figure 4:**
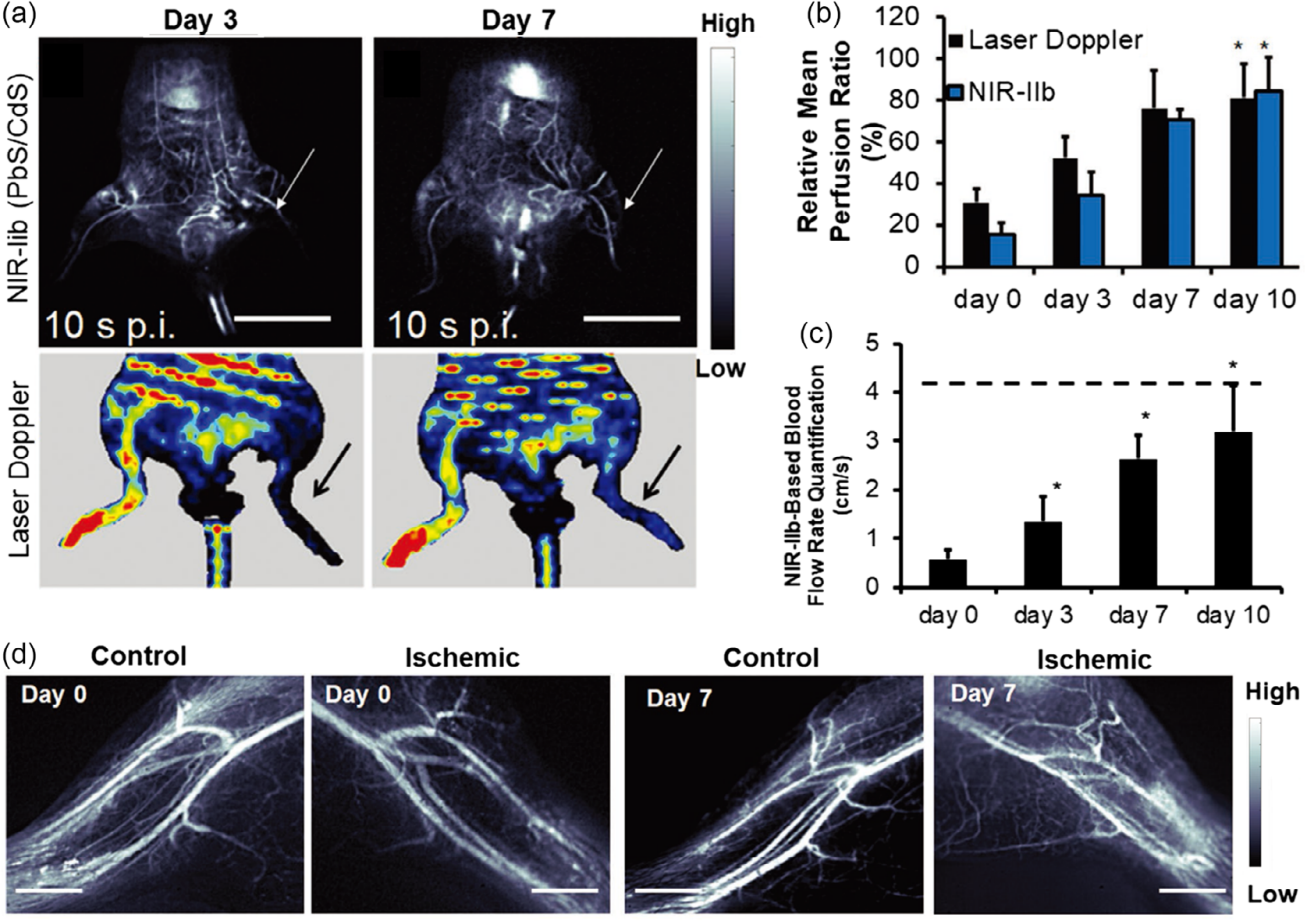
a) Upper: NIR‐II imaging of ischemic hindlimbs in mice. Fluorescence images both correspond to the time point of 10 s postinjection (p.i.). Scale bar: 2 cm. Lower: Representative laser Doppler spectroscopy images of ischemic limbs on days 3 and 7, respectively. b) Comparison of relative mean blood perfusion recovery in the ischemic limb by laser Doppler spectroscopy and NIR‐II fluorescence imaging. c) NIR‐II‐based quantification of blood flow rate. The dotted line denotes the mean flow rate of the control nonischemic limb. The arrow points to the ischemic limb (*p < 0.05, compared to day 0). d) Fluorescence images of mouse hind limb vasculature after induction of hind limb ischemia. Scale bar: 2 mm. Reproduced with permission.^[^ ^62^ ^]^ Copyright 2018, Wiley‐VCH.

PbS QDs with strong NIR‐II emission possess a variety of advantages for vascular imaging, including high PL QY (up to 60%), a narrow bandgap, and wide emission spectra covering the NIR window.^[^ ^59^ ^]^ Owing to possible surface oxidation in aqueous solutions, a doping strategy, antioxidant surface coating, or appropriate ligand modification are typically adopted to adjust the properties of PbS QDs, which could not only enhance their photostability but also reduce Pb^2+^ release to improve their biosafety. For example, Zhang et al. synthesized the Zn‐doped PbS QDs, which exhibited strong and stable NIR‐II emission. The Zn dopants effectively improved the PL QY of the QDs (up to 50%), prolonged the photoluminescence lifetime, and enhanced the thermal stability of PbS QDs. In NIR‐II optical imaging studies in vivo, the tiny cerebral vessels were successfully identified by PEGylated Zn‐doped PbS QDs.^[^ ^60^ ^]^ As an alternative to this cation‐doping strategy, Dai et al first coated a CdS shell onto PbS QDs, which protected the surface of the PbS core from oxidation. The amphiphilic polymer oleyamine‐branched polyacrylic acid (OPA) was then further coated onto the surface of PbS@CdS QDs with the branched PEG outer layer prolonging the blood half‐life of this material. The resultant QDs with ≈1600 nm NIR emission under 808 nm excitation allowed for the fast, real‐time imaging of blood flow at frame rates of up to 60 frames per second (fps) in vivo.^[^ ^61^ ^]^ In the subsequent study, by using similar PbS@CdS QDs, the authors were able to quantify vascular hemodynamics and perform high‐resolution imaging of vascular anatomy in mice with experimentally induced peripheral arterial disease. By using NIR‐II fluorescence imaging, the blood perfusion recovery and compensatory angiogenesis in the ischemic limb were monitored in real time (**Figure** **4**).^[^ ^62^ ^]^ In another work, Qian et al. employed a SiO_2_ shell to protect the PbS@CdS QDs from oxidation and further adopted the FDA‐approved Pluronic F‐127 (F‐127) polymer as a surface ligand to render the QDs water soluble and biocompatible. The resulting PbS@CdS@SiO_2_@F‐127 nanomaterials possessed good photostability and chemical stability. Through NIR‐II optical imaging, vascular structures in the brain can be visualized in 3D with high contrast and large spatial resolution can be acquired after intravenous injection of the nanomaterials. In addition, the cerebral blood flow of the mice can be monitored through real‐time video.^[^ ^63^ ^]^ Very recently, Chen et al. introduced the ribonuclease A (RNase A) protein as a biomolecular templating agent for preparing PbS/ZnS QDs in an aqueous phase to enhance their thermal stability and reduce their cytotoxicity. The resulting RNase A@PbS/ZnS QDs exhibited satisfactory photostability and biocompatibility, which can resist signal attenuation caused by thermal accumulation, allowing for long‐term imaging. Through these QDs, the temporal evolution of angiogenesis and vascular re‐establishment during the flap transplantation process can be monitored over a long‐term period, which is of great significance in clinical surgery.^[^ ^64^ ^]^ Very recently, Dai et al. synthesized water‐soluble PbS/CdS QDs with emission peaks at ≈1880 nm, which were adopted for in vivo imaging at wavelength window over 1700 nm with superconducting nanowire single‐photon detectors (SNSPDs). Under excitation with 1650 nm light, it is possible to perform in vivo molecular imaging of high endothelial venules with diameters as small as ≈6.6 μm, as well as immune cells in the lymph nodes.^[^ ^65^ ^]^

Apart from PbS QDs, Ag‐based QDs, including Ag_2_S, Ag_2_Se, and Ag_2_Te QDs, are a class of heavy metal‐free QDs that also exhibited NIR‐II emission. Although the PL QY of Ag‐based QDs still needs to be improved, Ag‐based QDs typically offer superior biocompatibility than PbS QDs.^[^ ^66^ ^]^ In 2013, Wang and co‐workers developed an in vivo dynamic imaging strategy using PEGylated Ag_2_S and Ag_2_Se QDs with NIR‐II emission. These QDs exhibited high colloidal stability in aqueous solution, as well good photostability, and offered higher spatial resolution for deep tissue imaging in comparison with the classic NIR‐I fluorescent dye, indocyanine green (ICG). Upon 808 nm laser irradiation, the lymphatic drainage and the angiogenesis of subcutaneous tumors can be visualized, and the anatomical structures of superficial vessels as thin as ≈100 μm can be depicted in mice with high spatiotemporal resolution in vivo.^[^ ^67^ ^]^ In subsequent studies, they passivated the Ag_2_Te QDs by overgrowing the Ag_2_S shell to construct a core–shell structure in order to further enhance the photoluminescence properties of QDs. The resultant Ag_2_Te@Ag_2_S QDs exhibited improved photoluminescence and stability in comparison with Ag_2_Te QDs. Through intravenous administration, QDs can delineate the vascular systems of mice with high feature fidelity, especially the thin sub‐branch vessels, under 808 nm light irradiation.^[^ ^68^ ^]^

### 3.2 Diagnosis of Atherosclerotic Plaque and Thrombosis

Atherosclerosis is a chronic progressive disease characterized by the deposition of excessive cholesterol in the arterial intima, which often results in complex lesions or plaques that protrude into the arterial lumen. With the continued accumulation of plaque, the possible rupture of vulnerable plaques may increase the adhesion and accumulation of platelets, forming thrombi, and leading to vascular obstruction.^[^ ^69^ ^]^ Even worse, the detached atheromatous debris may further block the distal vessels randomly, leading to acute life‐threatening events, such as myocardial infarction and ischemic stroke.^[^ ^70^ ^]^ Even though the plaque rupture may cause sudden and fatal emergencies, the formation of atherosclerotic plaques is a slow process, usually lasting for several years, providing ample opportunity for presymptomatic diagnosis.^[^ ^71^ ^]^ Therefore, advanced strategies for the early monitoring of atherosclerotic plaques are of great clinical significance because the timely and appropriate intervention can prevent disease progression, effectively preventing the development of plaque.

**Figure 5:**
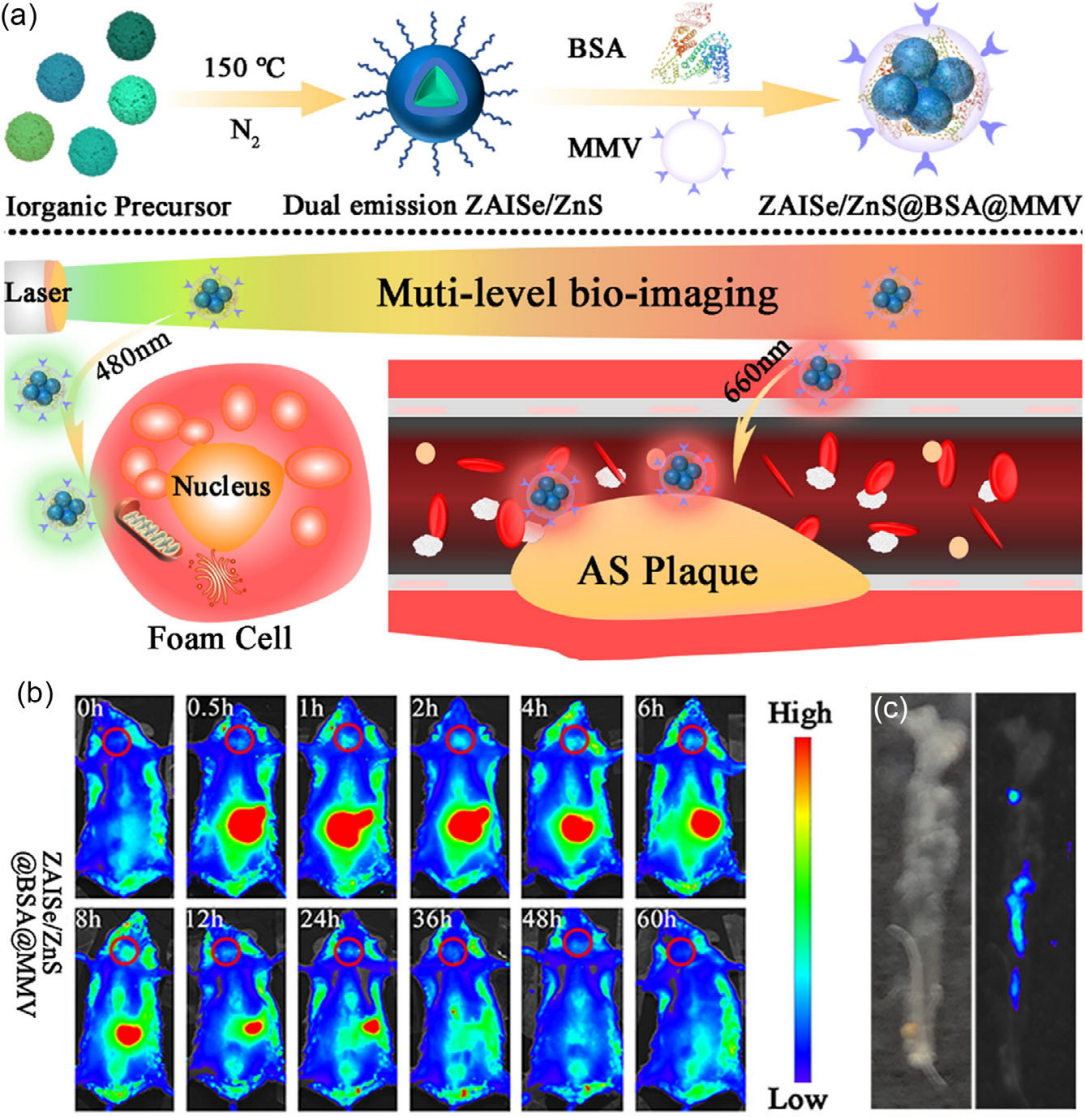
a) Illustration of the biomimetic dual‐emission ZAISe@ZnS@BSA@MMV preparation and its application for atherosclerotic plaque imaging. b) The typical NIR fluorescence images of ZAISe@ZnS@BSA@MMV in atherosclerotic plaque imaging at different time points. c) A bright field image (left) and fluorescent image (right) of resected aorta tissue after in vivo imaging. Reproduced with permission.^[^ ^75^ ^]^ Copyright 2023, Elsevier.

Currently, several clinical imaging modalities are widely used for plaque detection in patients with cardio‐cerebrovascular diseases, or in preclinical models, such as ultrasound imaging and MRI.^[^ ^72^ ^]^ However, they are either limited in spatiotemporal resolution or sensitivity. In comparison, optical imaging offers a more suitable imaging modality for depicting the location and progression of the plaques by using targeting nanomaterials with bright fluorescent signals.^[^ ^73^ ^]^ Semiconductor QDs have been exploited for the precise detection of atherosclerotic plaques. For example, in 2009, Haselton et al. modified QDs emitting at different emission wavelengths with the cell‐penetrating peptide maurocalcine through streptavidin‐biotin binding. The resultant QDs were adopted to label different leukocytes, including monocytes, macrophages, and T cells. After injection, owing to the recruitment of these QD‐labeled leukocytes, the fluorescence at different wavelengths was visualized in aortic lesions in the ex vivo tissues from the ApoE^−/−^ mouse model of atherosclerosis, which provided a feasible approach for investigating the role of distinct circulating leukocyte subsets in plaque development and progression.^[^ ^74^ ^]^ Very recently, Yu et al. coated nontoxic I‐III‐VI‐based quaternary ZnAgInSe (ZAISe) QDs with ZnS shells for surface passivation. The resulting ZAISe@ZnS core–shell QDs were further encapsulated by BSA to enhance water solubility and biocompatibility and were finally coated with macrophage‐derived microvesicles (MMV) to endow them with the ability to target sites of inflammation. Through in vivo fluorescent imaging, QDs were shown to specifically target carotid atherosclerotic plaque in mice models after intravenous injection, indicating the location of plaque with a strong fluorescent signal (**Figure** **5**). As a result, this QD‐based imaging strategy offers new possibilities for the early diagnosis of atherosclerosis.^[^ ^75^ ^]^

In addition to simply detecting the location(s) of plaques, QD‐based optical techniques may be able to noninvasively distinguish vulnerable atherosclerotic plaques from stable plaques in vivo by targeting distinct cellular and molecular epitopes. In vitro studies have demonstrated that modifying the QD surface with targeting moieties can endow them with the ability to bind to specific cellular epitopes that are abundant in vulnerable plaques.^[^ ^76^ ^]^ For instance, since a higher density of macrophages has been correlated with plaque instability, Mn‐doped Si QDs coated with dextran sulfate, which can target scavenger receptors on macrophages, may have the potential to readily detect vulnerable plaque.^[^ ^77^ ^]^

In 2008, Mulder et al. replaced the hydrophobic core of high‐density lipoprotein (HDL) with inorganic nanocrystals, including Au nanoparticles, Fe_3_O_4_ nanoparticles, and QDs, to produce novel contrast agents for molecular imaging. The resulting nanomaterials with a phospholipid corona were able to mimic the in vivo behavior of the endogenous HDL. Owing to the fact that HDL can remove cholesterol from macrophages in atherosclerotic plaques, these nanomaterials may have the potential to target and remedy atherosclerosis plaques, especially if they are vulnerable.^[^ ^78^ ^]^ This work displayed the prospects of QDs in the specific diagnosis of vulnerable plaque.

### 3.3 Diagnosis of Cerebrovascular Damage‐Related Diseases

The pathogenesis of various brain diseases, such as cerebral hemorrhage, traumatic brain injury, and ischemic stroke, involves cerebrovascular damage caused by different sources.^[^ ^79^ ^]^ Interestingly, QD‐based probes also possess the potential to visualize these cerebrovascular damage‐related diseases.

Cerebral hemorrhage is a generic term for a group of brain hemorrhagic diseases with high mortality rates, which can be mainly divided into two categories, i.e., intracerebral hemorrhage (ICH) and subarachnoid hemorrhage (SAH).^[^ ^80^ ^]^ ICH refers to the rupture of blood vessels in the brain parenchyma that causes blood to leak into the surrounding brain tissue. The etiology of ICH is closely associated with hyperlipidemia, diabetes mellitus, hypertension, aging, and other cerebral vascular diseases.^[^ ^81^ ^]^ SAH refers to the intracranial bleeding that extravasates into the subarachnoid space between the arachnoid mater and the pia mater of the brain. This category of disease is usually caused by cerebrovascular malformation and ruptured intracranial aneurysm in the base of the brain or under the tentorium cerebelli.^[^ ^82^ ^]^ The precise diagnosis of ICH and SAH is significant since emergent intervention and supportive treatment after onset, such as the reduction of intracranial pressure and the control of blood pressure/blood glucose, are necessary.^[^ ^83^ ^]^ In 2020, Joshi et al. reported a QD‐based in vivo NIR‐II imaging technique for tracking hyperglycemia‐induced ICH and blood–brain barrier (BBB) hyperpermeability in cerebral cavernous Malformation‐ (CCM) deficient mice (CCM1^+/−^). PEGylated Ag_2_S QDs with neutral surface charge and a long circulation half‐life were synthesized and employed as optical contrast agents. Through dynamic NIR‐II optical imaging, the murine cerebral vasculature was visualized, and the development of ICH and BBB impairment in hyperglycaemic CCM1^+/−^ mice was determined. This study demonstrated that NIR‐II fluorescence imaging with QDs may present an effective non‐invasive approach for detecting BBB disruption and visualizing cerebral hemorrhage with high sensitivity.^[^ ^84^ ^]^

Traumatic brain injury (TBI) is an abnormal alteration in brain function that is caused by an external force. This disease is a major cause of death and disability and is particularly prevalent in contact sports.^[^ ^85^ ^]^ Apart from the mechanical injury of the brain, secondary damage such as hemorrhage, inflammation, and ischemia can result in severe complications.^[^ ^86^ ^]^ In this context, the accurate diagnosis of primary brain injuries and the early prevention and/or treatment of secondary damage are crucial, especially for patients with mild symptoms after injury who may be misdiagnosed as well as patients with severe TBI who are in a coma.

**Figure 6:**
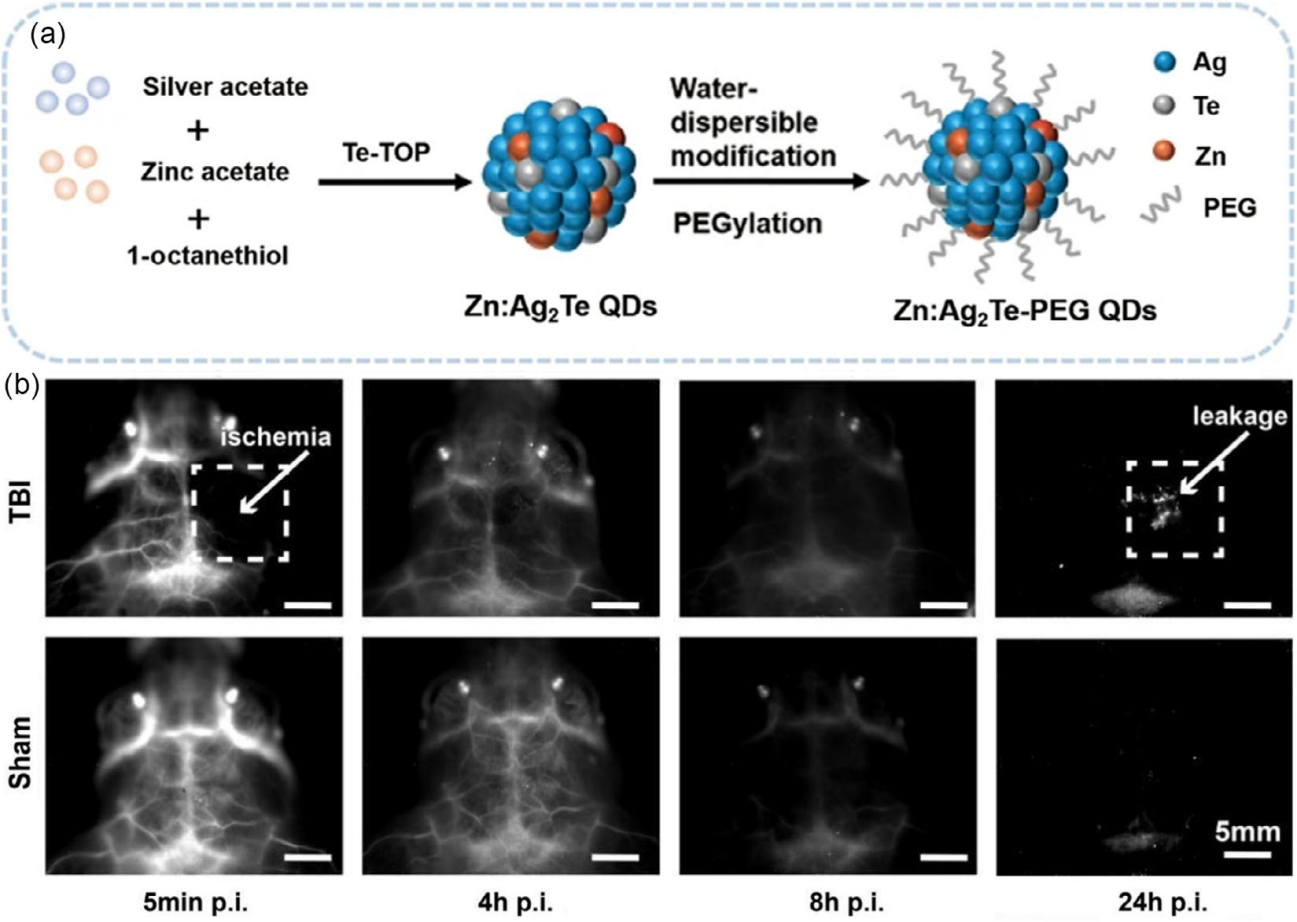
a) Schematic diagram of synthesized PEGylated Zn‐doped Ag_2_Te QDs. b) Dynamic monitoring of TBI mouse brain and sham injury mouse brain with QD‐enhanced NIR‐II imaging. Reproduced with permission.^[66c]^ Copyright 2022, Springer.

**Figure 7:**
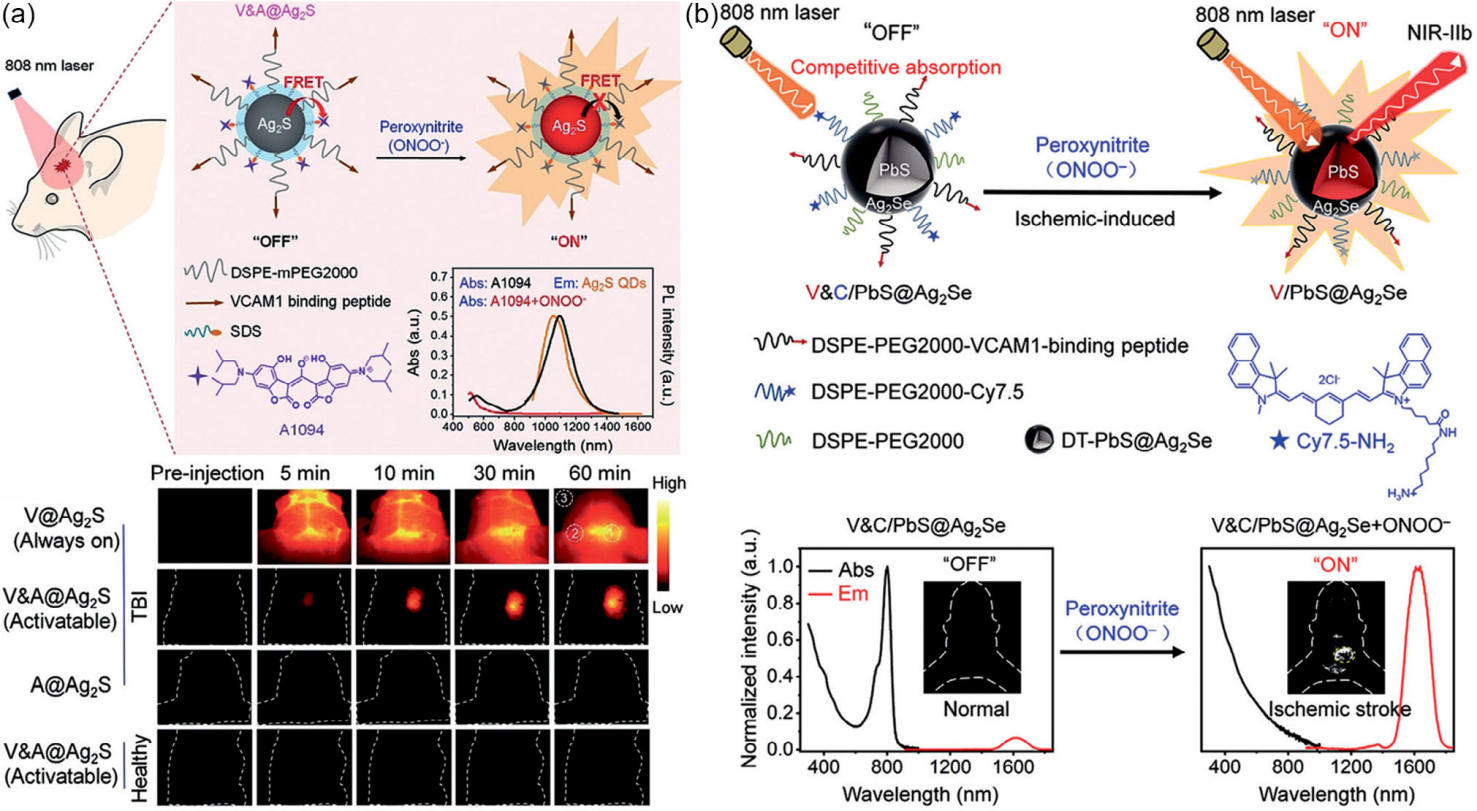
a) Preparation of the V&A@Ag_2_S probe and the timespan of NIR‐II fluorescence in brain vascular injury and healthy mice at different time points after injection of V@Ag_2_S, V&A@Ag_2_S, and A@Ag_2_S in vivo. Reproduced with permission.^[^ ^87^ ^]^ Copyright 2020, Wiley‐VCH. b) Schematic illustration of the construction of the V&C/PbS@Ag_2_Se nanoprobe and detection of ONOO^−^ in ischemic stroke mice. Reproduced with permission.^[^ ^92^ ^]^ Copyright 2021, Wiley‐VCH.

For the precise diagnosis of TBI, Cui and co‐workers synthesized PEGylated Zn‐doped Ag_2_Te QDs to enable noninvasive NIR imaging of murine cerebral vasculature with high spatial resolution. When administered intravenously, capillaries can be distinguished and, more importantly, the leakage of vessels and possible cerebral hemorrhage can be identified at 24 h post‐injection through the extravasation of the QDs in addition to the hypoperfusion region of TBI mice caused by the brain injury (**Figure** **6**).^[66c]^ Wang and co‐workers proposed a novel detection strategy for visualizing the biomarkers of TBI with NIR‐II QDs based on an activatable targeting nanoprobe, known as V&A@Ag_2_S. As reported, the reactive oxygen species (ROS) and reactive nitrogen species (RNS) are closely associated with the progression of TBI. Among these reactive species, peroxynitrite (ONOO^−^) originating from the injured cerebral vasculature can serve as a specific biomarker to construct nanoprobe. Following this design concept, the structure of V&A@Ag_2_S nanoprobe can be divided into three moieties: a vascular cell adhesion molecule (VCAM‐1) targeting peptide, a NIR absorber A1094, and Ag_2_S QDs. In the absence of ONOO^−^, the NIR emission of nanoprobes was quenched due to the energy transfer from Ag_2_S QDs to A1094. In contrast, after intravenous administration in the photothrombotic stroke mouse model, the nanoprobes actively accumulated in inflamed vascular endothelial cells in the TBI lesion due to targeting by the VCAM1‐binding peptide. In this ONOO^−^‐enriched site, A1094 on the nanoprobes was oxidized to elminate fluorescence quenching, activating the 1616 nm emission of the Ag_2_S QDs to enhance the signal at the site of TBI lesions (**Figure** **7**a).^[^ ^87^ ^]^

In addition to their diagnostic function for TBI, QDs can also be used to treat TBI through appropriate material design. Very recently, Cui et al. proposed a new strategy for image‐guided therapy of BBB impairment in mice after TBI using Mn^2+^‐doped Ag_2_Te QDs. With bright NIR fluorescence, dynamic variations in the BBB, including the transient cerebral hypoperfusion and cerebrovascular damage after TBI, can be visualized with high spatiotemporal resolution. More importantly, the atomically dispersed Mn on the surface of these Ag_2_Te QDs endowed the nanomaterials with catalase (Cat)‐like and superoxide dismutase (SOD)‐like antioxidant activity to effectively remove H_2_O_2_, •O_2_^−^, and •OH from the local environment with high efficiency. In an experimen*t* testing, the benefits of this approach, the rapid reconstruction of the BBB, and a recovery of neurological function were achieved within 10 days after the onset of TBI under the treatment of QDs. Thus, this strategy has the potential benefit of using multifunctional QDs as effective theranostics of TBI.^[^ ^88^ ^]^

Ischemic stroke and its complications may lead to severe disabilities and cognitive deficits. The etiology of ischemic stroke can arise from the transient or permanent occlusion of cerebral vessels, which causes a decrease in blood flow to the brain and injuries to brain tissues and the central nervous system in general.^[^ ^89^ ^]^ The main treatment for ischemic stroke involves the breakup of the arterial occlusion and restoration of perfusion to reverse brain tissue ischemia. However, this treatment route can be only used in patients within several hours of stroke onset because the salvageable ischemic tissue will quickly turn into irreparable dead tissue as the stroke‐induced damage progresses.^[^ ^90^ ^]^ Additionally, improper thrombolysis does not show a benefit to patients and can even lead to inferior outcomes, such as hemorrhage.^[^ ^91^ ^]^ Considering that the status of stroke varies from patient to patient, the precise diagnosis of ischemic stroke is critical for determining the appropriate intervention.

Similar to TBI, after the onset of ischemic stroke, the occlusion of the blood vessels leads to inflammation and oxidative stress, thereby creating a large amount of ROS and RNS. Wang and co‐workers developed a NIR‐II QD‐based detection strategy for visualizing early ischemic stroke, following the design concept of the aforementioned “smart” stroke nanoprobes. More specifically, a VCAM1‐targeted peptide and the ONOO^−^‐responsive fluorophore, Cy7.5, were attached to the surface of PbS@Ag_2_Se QDs. In the absence of ONOO^−^, the emission of V&C/PbS@Ag_2_Se at 1616 nm was suppressed due to the competitive absorption of excitation irradiation between Cy7.5 fluorophores and PbS@Ag_2_Se QDs. In contrast, in the inflamed vascular endothelial cell within an ischemic lesion—and therefore rich with ONOO^−^—the Cy7.5 on the nanoprobes is oxidized, eliminating its competitive effect and thereby activating the 1616 nm emission of the QDs to delineate the ischemic lesions. This smart nanoprobe enabled the rapid in vivo diagnosis of early ischemic stroke with high spatial resolution (Figure 7b).^[^ ^92^ ^]^ In a recent work, Liu and co‐workers proposed an imaging strategy for both the diagnosis of the cerebrovascular structure and evaluation of hemodynamics in ischemic stroke and TBI in which the Ag_2_Se QD‐sensitized lanthanide‐doped nanocrystals (QDs‐LnNCs) with emission beyond 1500 nm were synthesized as optical contrast agents. With the introduction of Ag_2_Se QDs, the NIR‐II emission of LnNCs was dramatically enhanced, endowing the nanomaterials with in vivo vascular imaging potential. In an in vivo experiment, hypoperfusion and slowed blood flow can be clearly identified. In addition, the impaired BBB and potential hemorrhage can be visualized via the extravasation of QDs in the brain parenchyma of TBI models.^[^ ^93^ ^]^

Apart from stroke diagnosis, several semiconductor QDs with strong emission have been employed to label different types of stem cells, such as bone marrow stromal cells and neural stem cells, that were subsequently transplanted into the infarcted brain of animals to promote nerve regeneration and repair. The satisfactory fluorescent properties of QDs provided the possibility of tracing these grafted cells in vivo for several weeks after implantation.^[^ ^94^ ^]^

## 4 Microbial Infections

Infectious diseases and inflammation caused by microbial pathogens, including bacteria and viruses, remain a major global health threat, especially for immunodeficient populations.^[^ ^95^ ^]^ For bacterial infections, although antibiotics often have outstanding therapeutic effects, antimicrobial resistance has gradually become important and alternative treatments are urgently needed.^[^ ^96^ ^]^ For viral infections, chronic diseases caused by viruses are often more difficult to cure, and the efficiency of available antivirals and vaccines is limited by the rapid emergence of viral mutation.^[^ ^97^ ^]^ These challenges emphasize the need to improve our understanding of both microbial biology and canonical antimicrobials, including not only the understanding of resistance mechanisms and innovations in drugs and vaccines but also the accurate diagnosis of different disease etiology and the rational administration of antimicrobials.

In recent studies, QD‐based nanomedicines with intrinsic optical or magnetic properties, engineered targeting capabilities, and/or site‐specific release have provided a library for precisely detecting, effectively preventing, and specifically treating infections caused by a variety of microorganisms.

### 4.1 Diagnosis of Viral Infections

The spread of viral diseases is a major global concern. The high infectivity and mutation rate make it difficult to quickly and precisely detect viral infections. Among current detection methods, polymerase chain reaction (PCR) is an accurate diagnostic strategy that works by precisely detecting the target nucleic acid of viruses.^[^ ^98^ ^]^ However, it is difficult to perform point‐of‐care detection using this approach because of the equipment and technical training required. An enzyme‐linked immunosorbent assay (ELISA) is another detection method based on specific antigen–antibody interactions that can detect viral proteins or virus‐specific IgM and IgG in the serum of patients, but costly reagents are needed.^[^ ^99^ ^]^ To solve these problems, improved biomarker‐based biosensing technologies have been developed over the past few decades and used worldwide to detect viral infections.^[12a]^ Correspondingly, QD‐enabled biosensing techniques have also been reported. QDs can be a promising option for specific probes that identify biomarkers involved in viral infection processes, allowing them to directly diagnose viral infections in physiological components of patients through colorimetric, amperometric, impedimetric, fluorescence, and optomagnetic technology.^[^ ^100^ ^]^

One of the biggest challenges in detecting analytes in biological samples is the accurate capture of virus‐specific biomarkers that are not abundant. In this context, the unique properties of QDs, including their high surface‐to‐volume ratio, quantum size effects, high adsorption, and reactive capacity, that allow for sophisticated surface modification or immobilization, and the effective amplification of the signal may enable the development of ultrasensitive and highly selective labeled‐biosensing techniques with low detection limits. For example, in 2007, Chan et al. developed a diagnostic system for multiplexed, high‐throughput analysis of biomarkers in human serum samples through the combination of nano‐ and micro‐technologies (QDs and microfluidics). Two types of CdSe/ZnS QDs were synthesized and integrated into polystyrene beads at different ratios, resulting in three distinct QD barcode particles. The viral‐specific antigen, including hepatitis B surface antigen (HBsAg), HCV nonstructural protein 4 (NSP4), and HIV glycoprotein 41 (gp41), was covalently conjugated on the surface of QD‐barcoded particles. After incubating these particles with human serum that contained corresponding antibodies, the antibodies would be captured by the QD‐barcoded particles and fluorophore‐antibody conjugates were employed to target and detect the presence of the target antibodies. In particular, the narrow emission peak of QD‐enabled barcode‐based recognition to determine which of the three target antibodies exists.^[^ ^101^ ^]^ In another work, Gao and co‐workers coated CdTe QDs with denatured bovine serum albumin (dBSA) molecules. The resulting CdTe@dBSA can largely avoid nonspecific adsorption on cell membranes and can therefore serve as a potential platform to establish high‐sensitivity molecular probes for virus detection. On this basis, streptavidin was covalently conjugated onto the surface of CdTe@dBSA, which successfully endowed the QDs with specific binding affinity for the immunofluorescence‐based detection of anti‐Epstein–Barr virus (EBV) capsid antigen IgA (VCA‐IgA) in the serum of nasopharyngeal carcinoma patients.^[^ ^102^ ^]^

In the past three years, owing to the ever‐increasing demands in resisting and managing the coronavirus disease 2019 (COVID‐19) pandemic, the development of rapid, highly sensitive, and accurate tools for diagnosing the viral load present in populations has become an urgent challenge.^[^ ^103^ ^]^ With this motivation, researchers have developed QD‐based lateral flow immunoassays (LFIA) to replace the traditional gold‐based LFIA and improve the detection sensitivity of viral surface proteins or the antibodies against virus while reducing cost. Specifically, the detection of antibodies can provide information about if someone has been vaccinated or infected previously, while the assays for viral protein will reflect the active infections. Xiong et al. reported a CdSe@ZnS QD nanobead‐based LFIA (QB‐LFIA) for detecting SARS‐CoV‐2 antibodies level in serum. The QB‐LFIA adopted a double‐antigen sandwich immunoassay format. The expressed recombinant SARS‐CoV‐2 spike proteins immobilized on a test (T) line are used to capture the target antibodies in the serum, which can further conjugate with the fluorescent QBs to be detected. Using this strategy, the researchers showed that the QB‐LFIA exhibited an approximately one order of magnitude improvement in sensitivity compared with the commercial gold‐based LFIA.^[^ ^104^ ^]^ Similarly, Hu and co‐workers developed CdSe@CdS@ZnS QDs‐based reporters consisting of dendritic silica templates, hydrophobic QDs, silica‐encapsulating layers, and hydrophilic polymer layers. The fluorescent signals were effectively amplified by the compact 3D assembly of QDs. Through using the dual‐antigen sandwich format, over 300 negative and 97 positive specimens were accurately diagnosed with high sensitivity and specificity, confirming the commercial PCR and chemiluminescence immunoassay results.^[^ ^105^ ^]^

**Figure 8:**
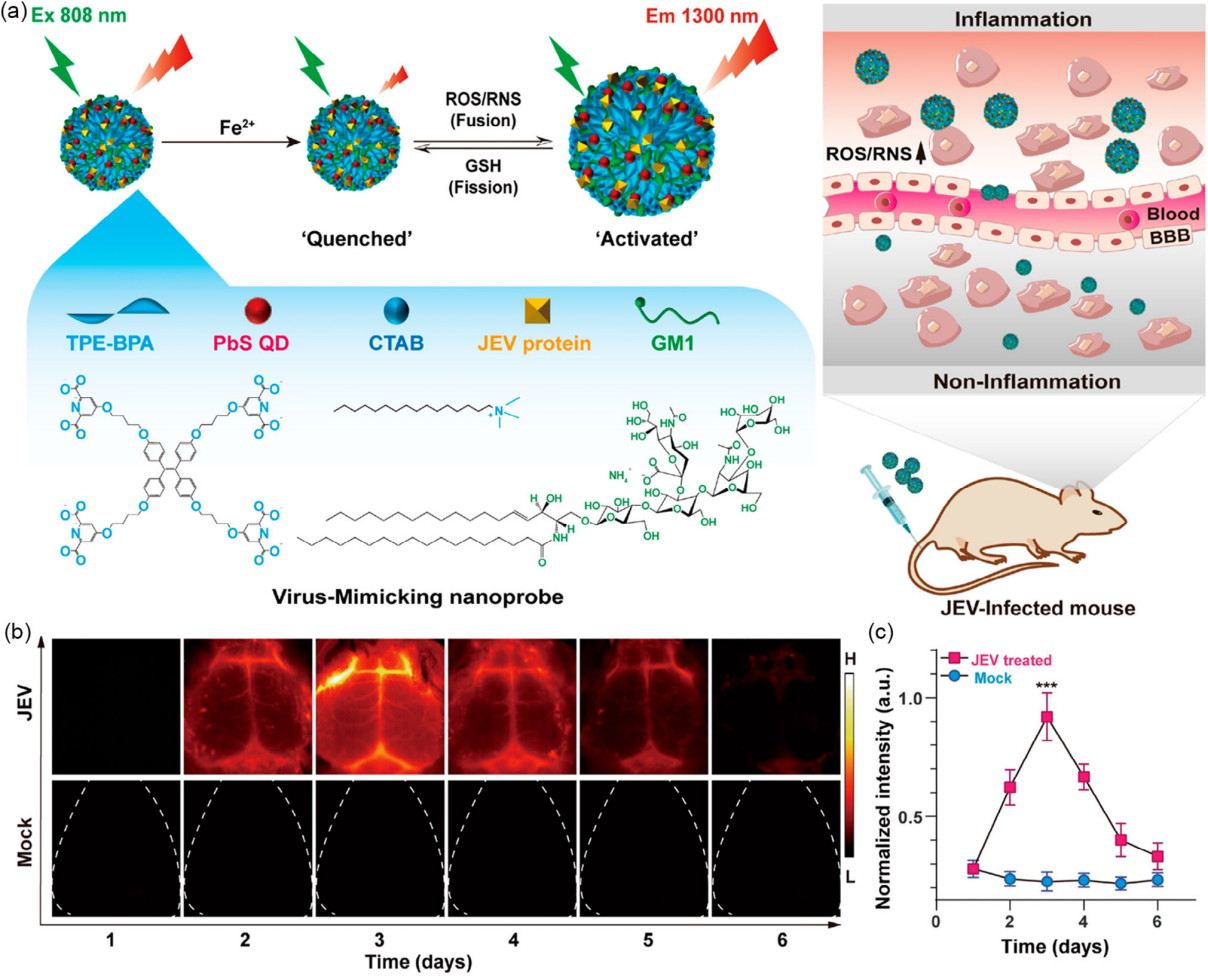
a) Schematic of the QDs&Fe^2+^@VVesicle composition and mechanism of ROS/RNS detection in vivo. b) Time‐dependent NIR‐II fluorescence images of JEV‐ and mock‐infected mice after intravenous injection of QDs&Fe^2+^@VVesicles. c) Quantification of the image intensities in frame b). Reproduced with permission.^[^ ^108^ ^]^ Copyright 2022, Wiley‐VCH.

Apart from in vitro biosensing for diagnosis, QD‐based probes with high functionality, targeting specificity, and chemical stability can be also designed for sensitively imaging viral diseases in vivo. In comparison with in vitro assays, in vivo imaging can provide more adequate diagnostic information for viral diseases, such as the location and the severity of infection, or even provide a prognosis. In 2014, Cai et al. reported a QD‐based virus labeling strategy for tracking viral respiratory infections in vivo. They labeled avian influenza H5N1 pseudotyped virus (H5N1p) with NIR‐emitting CdTeSe/ZnS QDs via bioorthogonal chemistry. The QD‐labeled H5N1p exhibited bright photoluminescence and photostability, which enabled the real‐time noninvasive tracing of viral infections in mice. In addition, the severity of viral infection was able to be quantitatively analyzed by measuring the signal intensity and Cd concentration in different tissues. These experiments revealed that the in vivo dynamics of QD‐H5N1p infection correlate closely with the administration of the antiviral agent oseltamivir carboxylate and mouse antiserum. This study demonstrated the potential application of QD‐labeled strategies for antiviral drug evaluation.^[^ ^106^ ^]^ In 2021, Pan et al. added specific antisense oligonucleotides (ASOs) to the surface of PbS QDs through a click reaction to endow QDs with SARS‐CoV‐2 RNA‐targeting ability. Once one ASO strand on the surface of QDs hybridized with the target viral RNA strand, the remaining ASO strands would attract other RNA strands to accumulate the RNA of SARS‐CoV‐2 and induce QD aggregation, thereby activating NIR‐II emission. This virus‐triggered NIR‐II emission has been used for imaging COVID‐19 infections in murine lung tissue.^[^ ^107^ ^]^ Very recently, Zhao et al. reported an activatable nanoprobe for the real‐time monitoring of ROS/RNS levels in the process of viral infection. PbS QDs with 1300 nm NIR‐II emission were encapsulated with the Fe^2+^‐coordinated Japanese encephalitis virus (JEV)‐mimicking vesicle, known as QDs&Fe^2+^@VVesicle. The incorporation of a viral envelope (E) protein on the vesicle surface enabled the nanoprobe to cross the BBB. In viral infection‐induced inflammatory regions, increased ROS/RNS will oxidize Fe^2+^ to induce the fusion of vesicles, thereby activating the QD emission. Through this responsive characteristic, the nanoprobes were employed to provide real‐time monitoring of dynamic ROS/RNS concentrations during the progression of viral encephalitis. This QD‐based smart nanoprobe can be regarded as an appropriate imaging agent that allows for optical imaging of viral infections in vivo and provides a new tool for studying the temporal evolution of viral encephalitis (**Figure** **8**).^[^ ^108^ ^]^

### 4.2 Diagnosis and Treatment of Bacterial Infections

In addition to viral infections, bacterial infections, especially multidrug‐resistant bacterial infections, are another category of clinical diseases with high incidence and mortality rates, which have become a substantial threat to human health and a substantial economic burden on families and society.^[^ ^109^ ^]^ In addition to traditional approaches, advanced QD‐based functional nanomaterials have been developed in recent years, offering additional options for the diagnosis and treatment of bacterial infections.

Rapid and precise detection of pathogenic bacteria is a key prerequisite for enacting appropriate clinical interventions. Benefitting from the bright and intensive fluorescence caused by the quantum size effect and the dielectric constraint effect, several semiconductor QD‐based fluorescence sensing techniques have been developed for bacteria detection, similar to the aforementioned QD‐based biosensing of viruses.^[^ ^110^ ^]^ For example, Cai et al. developed a ZnCuInSe QD‐based fluorescence sensor for the classification, quantification, and imaging of *Staphylococcus aureus*. Because Gram‐positive bacteria only possess a cytoplasmic membrane covered by a loose and poriferous cell wall, whereas Gram‐negative bacteria possess an additional outer membrane, GSH‐modified, water‐soluble QDs can be used to selectively bind with Gram‐positive bacteria, enabling the classification of the bacteria. Leveraging this property, QDs can be employed to detect *S. aureus* with high sensitivity.^[^ ^111^ ^]^ In another work, Wang et al. developed a fluorescent LFIA strip for bacterial detection by using magnetic‐core@dual QD‐shell nanoparticles (Fe_3_O_4_@DQDs) as multifunctional fluorescent labels. More specifically, the antibody‐modified Fe_3_O_4_@DQDs were designed to capture bacteria from samples and be collected through magnetic separation. Thereafter, CdSe@ZnS QDs provided a strong fluorescent signal to brighten the test line of the antibody‐bacteria‐antibody sandwich‐like immunocomplexes as they are formed. Through this method, *Streptococcus pneumoniae* in the complex blood sample was detected with high sensitivity, indicating that this method has promise for bacterial testing in clinical diagnosis.^[^ ^112^ ^]^ Recently, Zhang et al. proposed a QD nanobead‐labeled LFIA, which can be used to quantitatively detect *Salmonella typhimurium* (ST) when combined with strand displacement loop‐mediated isothermal amplification (SD‐LAMP). Benefiting from the strongly emissive CdSe/ZnS QDs, the resultant nanobeads served as fluorescence reporters, and ST was quantitatively detected by analyzing the fluorescence intensity of the test (T) line and control (C) lines. This method has the potential to be employed for rapid bacterial detection in foods for food security or for clinical diagnosis.^[^ ^113^ ^]^

In addition to in vitro biosensing applications, in vivo imaging strategies for the diagnosis and evaluation of bacterial infections have been also developed to guide the prescription of antibiotics. For example, Chen et al. developed a PbS QD‐based NIR‐II fluorescence imaging strategy for the real‐time monitoring of bacterial infections in vivo, thereby optimizing the timing of antibiotic administration. Specifically, after being capped by RNase‐A enzymes, PbS QDs exhibited satisfactory labeling capability on four bacterial strains tested, including *S. aureus*, *S. epidermidis*, *E. coli*, and *S. anginosus*. In subsequent in vivo imaging experiments, the temporal evolution of fluorescence intensity of QDs at infection sites reflected the infection recovery process. This real‐time monitoring of bacterial infection possesses is potentially useful for directing the administration of antibiotics over the appropriate time window.^[^ ^114^ ^]^

Clinically, antibiotics are the most widely used treatment for bacterial infections due to their ability to effectively kill bacteria and save the lives of patients. However, the long term or frequent use of antibiotics can quickly promote the evolution of drug resistance.^[^ ^115^ ^]^ In comparison with antibiotic‐sensitive bacteria, antibiotic‐resistant bacteria more readily spread between people as first‐line antibiotics are rendered ineffective. Therefore, new approaches to fight bacterial infections are an urgent clinical need. In some cases, QD‐based treatment strategies for bacterial infection have been exploited to address this issue.

**Figure 9:**
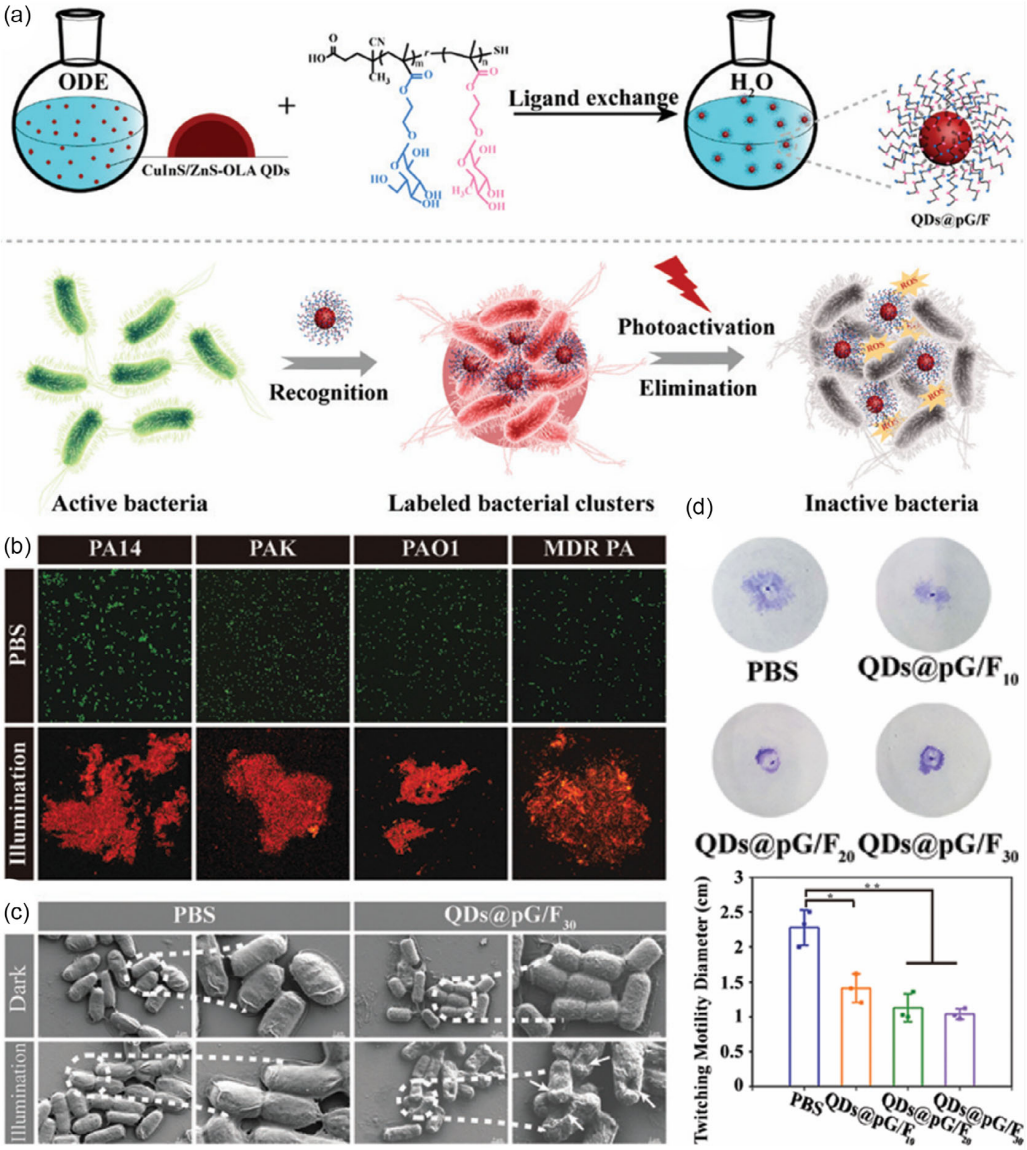
a) Preparation and antimicrobial application of QDs@pG/F. b) Fluorescence micrographs of four strains of *P. aeruginosa* (i.e., PA14, PAK, PAO1, and MDR PA), labeled with acridine orange (green, live bacteria) and ethidium bromide (red, bacteria with damaged membranes), after incubation with QDs@pG/F in the dark or when illuminated. c) Scanning electron microscope images of PA14 under different conditions: PBS (dark), PBS (illumination), QDs@pG/F30 (dark), and QDs@pG/F30 (illumination) and magnified views from the dashed circular frames. d) Photographs and quantified results of the twitching motility of PA14 with and without nanobiocides. Reproduced with permission.^[^ ^117^ ^]^ Copyright 2021, the Royal Society of Chemistry.

Unlike current antibiotics, the antibacterial mechanisms underlying QDs can mainly be attributed to their ability to generate ROS upon photoexcitation. Because ROS can kill the bacteria in a nonspecific manner, the known drug resistance mechanisms can largely be avoided. Therefore, the specific delivery of ROS‐generating QDs to the inflammatory site of bacterial infection is a promising strategy as a replacement or supplement to the administration of traditional antibiotics. Chatterjee et al. proposed a new strategy for treating drug‐resistant bacterial infections based on indium phosphide (InP) QDs. These InP QDs were 3.2 nm in diameter and tuned to a conduction band beyond the −0.33 V reduction potential of dissolved oxygen to superoxide. Upon NIR light irradiation, the photoactivated QDs generated toxic superoxide, thereby killing drug‐resistant bacteria in vivo and reducing subcutaneous abscess infection in mice. Therefore, these InP QDs appear to be a promising alternative to conventional antibiotics.^[^ ^116^ ^]^ In the subsequent studies, the group further demonstrated that CdTe QDs with a 2.4 eV bandgap can also be activated by blue light to produce the toxic ROS, thereby treating the subcutaneous infection of mice.^[13a]^ By employing a similar design concept, Zhang et al. developed CuInS/ZnS QDs to eliminate the pathogenic, multidrug‐resistant *P. aeruginosa*. Hetero‐multivalent glycomimetics were coated on the surface of these QDs, which enhanced the biocompatibility of the resulting QDs@pG/F and endowed them with bacteria‐capturing capabilities. Under the irradiation at 660 nm, the QDs@pG/F were activated and produced ROS, enabling the disruption of bacterial adhesion and inhibition of biofilm formation. This study demonstrated the potential of QDs to fight against drug‐resistant bacteria while allowing for the possibility of visualized diagnosis (**Figure** **9**).^[^ ^117^ ^]^

## 5 Medical Tattoos

Tattoos are a common form of body adornment, which are ubiquitous among populations around the world. Tattoos are often used as a form of self‐expression with artistic, spiritual, or other meanings. In addition to ornamental purposes, tattoos have also been employed for medical applications over the past few decades.^[^ ^118^ ^]^ For example, tattoos can be used to conceal scars or other defects. In addition, tattoos have also been used to label the locations of lesions for guiding the upcoming surgery or repeated treatments.^[^ ^119^ ^]^ Furthermore, tattoos can also be used to record medical information. For the convenience of first aid, blood‐type tattoos have been used for both civilians and military personnel.^[^ ^120^ ^]^ Similarly, it has been suggested that tattoos could be used as a reliable method for permanently recording children's immunization status.^[^ ^121^ ^]^

Nevertheless, medical tattoo technologies still face many problems. Traditional tattoos are applied through the repeated injection of pigments or inks into the skin using needles or lancets, which may lead to pain, bleeding, and/or infection. In addition, tattoo inks may cause allergic reactions, hypersensitization, and inflammation due to impurities, which may include heavy metals (e.g., Cr, Cd, and Hg), primary aromatic amines, polycyclic aromatic hydrocarbons, formaldehyde, parabens, and isothiazolinones. These adverse reactions may present acutely or only become apparent weeks, months, or years after the tattoo was applied.^[^ ^122^ ^]^

In recent years, nanotechnology has provided a feasible alternative to traditional tattoo inks. The biocompatibility and physicochemical stability of nanomaterials allow for the continuous and long‐term persistence of tattoo patterns.^[^ ^123^ ^]^ Furthermore, the replacement of traditional tattoo inks with smart nanomaterials even opens up the possibility of monitoring interstitial biomarkers in real time.^[^ ^124^ ^]^

Apart from new nanomaterial‐based inks, the administration route of tattoos has the potential to be improved with the development of novel delivery approaches. Advanced administration methods used to deliver drugs can even be adopted for medical tattoo administration. In particular, microneedle patches have been developed as an alternative to traditional injections that allow simple administration of various tattoo inks. In comparison with traditional injection methods, an array of sharp, microscale needles with appropriate length can pierce the stratum corneum while avoiding nerves or damaging dermal blood vessels, resulting in painless access to dermal layers.^[^ ^125^ ^]^ In addition, the tiny and shallow wounds resulting from microneedle penetration can heal on the order of minutes to hours, which largely enhances patient compliance since it reduces the likelihood of infection.^[^ ^126^ ^]^

**Figure 10:**
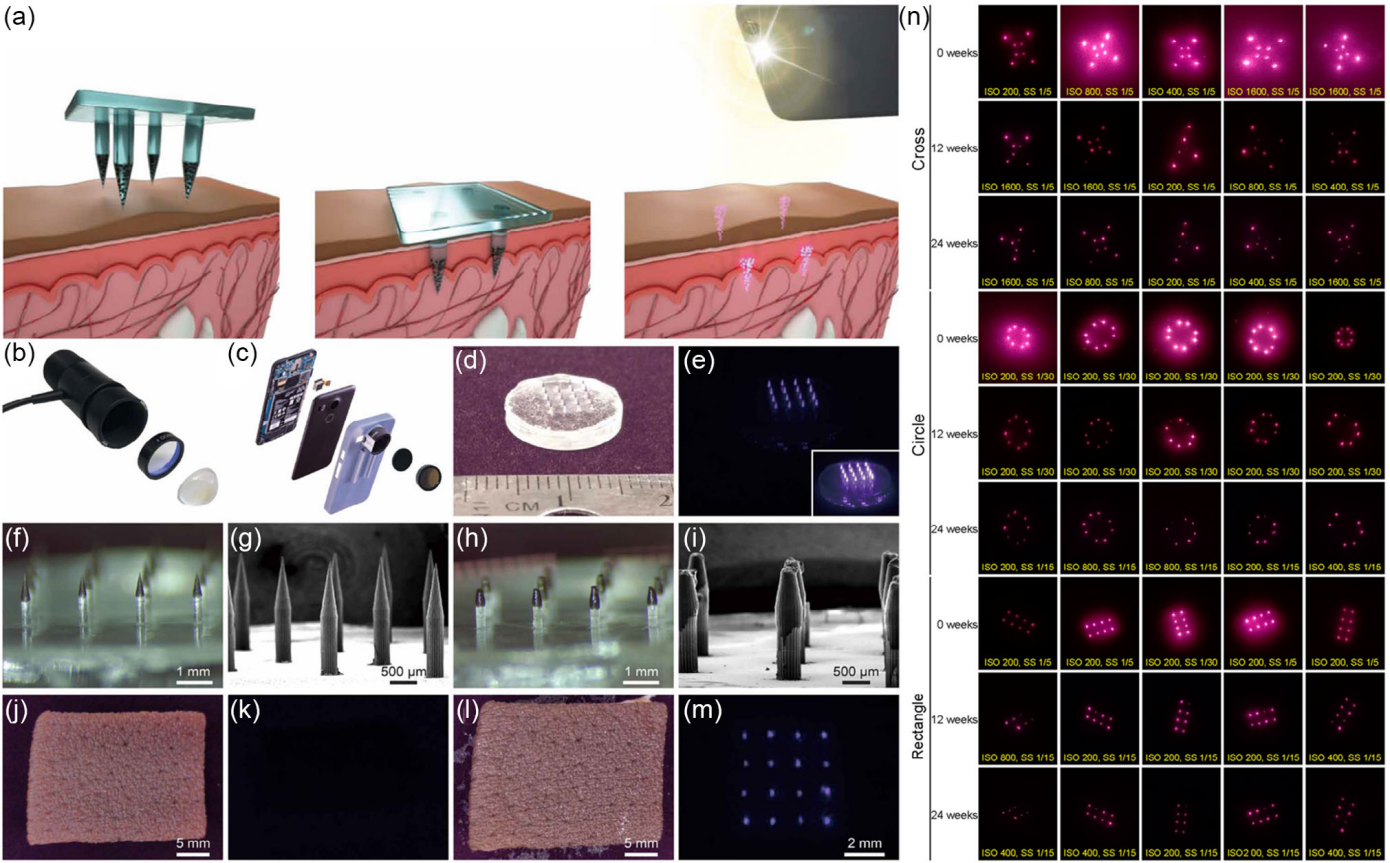
a) PMMA‐encapsulated QD‐based fluorescent microparticles are distributed through an array of dissolvable microneedles in a distinct spatial pattern. Microneedles are then applied to the skin for 2 to 5 min, resulting in partial dissolution of the microneedle and retention of fluorescent microparticles. An NIR LED and an adapted smartphone are used to image patterns of fluorescent microparticles retained within the skin. b) Photograph of disassembled LED used for NIR illumination at 780 nm combined with an 800‐nm short‐pass filter and aspheric condenser. c) Photograph of disassembled NIR imaging smartphone with two external 850‐nm long‐pass filters set in a 3D‐printed phone case. Images of a 16‐needle microneedle patch containing PMMA‐encapsulated QDs were collected with the adapted smartphone under ambient indoor lighting d) without and e) with the pair of 850‐nm long‐pass filters under LED illumination. Inset shows an image at a higher exposure. f,h) Optical and g,i) SEM images of microparticle‐loaded microneedles f,g) before skin application and h,i) after administration to explanted pig skin. Adapted smartphone images of pigmented human skin before microneedle application j) without and k) with the 850‐nm long‐pass filters. Smartphone images of human skin after application l) without and m) with the 850‐nm long‐pass filters. n) Cropped, but otherwise raw, smartphone images collected from a fixed distance showing the intradermal NIR signal from PMMA‐encapsulated QDs delivered via microneedle patches on rats 0, 12, and 24 weeks after administration. Reproduced with permission.^[^ ^14^ ^]^ Copyright 2019, American Association for the Advancement of Science.

Through using both of these two advanced technologies, Langer and co‐workers proposed a novel strategy to encode medical history in the dermis using the specific spatial patterning of QDs in the dermis. Biocompatible CuInSe_2_@ZnS:Al QDs with strong NIR emission were loaded into nondegradable poly(methyl methacrylate) microparticles and further embedded into dissolvable microneedle patches. After being delivered into the dermis of live rats or explanted human or pig skin, the specific spatial patterns with NIR emissions were invisible to the naked eye but detectable using an inexpensive, modified smartphone (**Figure** **10**). The distinct spatial distribution of QDs in the skin could ultimately be used to encode different medical information, such as an on‐patient record of an individual's vaccination history. Benefiting from the outstanding photobleaching resistance of QDs, the NIR patterns applied to the dermis of rats were still identified 9 months after administration through using a machine learning algorithm. The subsequent histological analysis revealed that the retained particles showed favorable biocompatibility, similar to largely inert PMMA‐based tattoo dyes, which do not trigger severe foreign body reactions. This invisible QDs‐based medical tattoo technology provides a new approach for medical data recording and biosensing applications with the potential to improve healthcare management.^[^ ^14^ ^]^ To date, several types of nanomaterials have been designed to serve as environmentally responsive inks for colored tattoos or tattoos only visible under ultraviolet light irradiation, thereby protecting potentially sensitive patient information. Nanomaterial‐based microneedle patch tattoos with distinct patterns, such as numbers, letters, or symbols, can be used to recode various medical information including medical alerts and vaccination status.^[^ ^127^ ^]^ When engineered to exhibit favorable biocompatibility and optical properties, QDs are an appealing candidate for use as novel tattoo inks capable of accurately recording large amounts of data on a patient with potential applications that include medical information storage, physiological monitoring, and cosmetic outcomes.

## 6 Conclusions and Future Perspectives

In this manuscript, we provide an overview of the state‐of‐art of semiconductor QDs that have been engineered for the diagnosis and treatment of various diseases. Benefiting from strongly emissive and tunable fluorescence that covers a broad spectral range from the visible into the near‐infrared region, QDs represent an appealing nanoplatform for disease theranostics in precision medicine. In addition to their favorable optical properties, magnetic properties can also be introduced by adjusting the chemical composition, nanostructure, and/or surface modification to enable multimodal imaging with MRI. Further, owing to their intrinsic defect‐induced nonradiative relaxation pathways and broad absorption wavelength, several categories of QDs exhibit photothermal and/or photodynamic therapeutic efficacy under light activation. In addition to the intrinsic properties, QDs can be coupled with different ligands or functional cargo molecules through surface modification to regulate pharmacokinetics and pharmacodynamics. The size range, emission maxima, toxicity information, and biomedical applications offered by each QD are summarized in **Table** **1**. Overall, with both intrinsic and acquired properties, semiconductor QDs have the potential to become all‐in‐one nanoplatforms for a variety of biomedical applications.

**Table 1:** Summary of the characteristics, toxicity information, and medical applications of representative QDs

Material	Size range [nm]	Emission maxima [nm]	Toxicity information	Medical applications	Ref.
CdSe@ZnS	5	630–640	RES uptake in liver and spleen	Visible optical tumor imaging	[21, 22]
Zn‐Cu‐In‐Se@ZnS	4.1	630–800	Low cytotoxicity	Multiplexed visible and NIR‐I fluorescent mapping of biomarkers of tumor cells in vitro	[25]
Ag_2_S	5.4	≈1200	Cleared through biliary pathway	NIR‐II optical tumor imaging	[30]
Ag_2_S	–	1200	No obvious organ damage and toxicity	Preoperative NIR‐II optical diagnosis and image‐guided brain tumor resection	[31]
Ag_2_S	5.7	1000–1400	Low cytotoxicity	NIR‐II optical tumor imaging	[32]
CuInS_2_@ZnS:Mn	3.6	620	Low cytotoxicity; part of particles cleared through renal pathway	Visible optical/MR tumor imaging	[34]
Ag_2_Se@Mn‐^64^Cu	1.9	750	Renal clearance; no obvious organ damage and toxicity	NIR‐I optical/MR/PET tumor imaging	[35]
PbS	6.9	1600	Cleared through biliary pathway	NIR‐II optical tracking vaccine dynamics in immunotherapy	[36]
CdSe@ZnS@SiO_2_ & InP@ZnS@SiO_2_	–	–	Low cytotoxicity; no obvious hemolysis	Visible/NIR‐I optical tracking vaccine dynamics in immunotherapy	[37]
B	2‐3	–	No obvious organ damage and toxicity	Photoacoustic imaging and PTT of tumor	[42]
CoS_*x*_	5.8 for CoS_2_	–	Low cytotoxicity; no obvious organ damage and toxicity	PDT/PTT of tumor	[43]
CuInSe_2_@ZnS:Mn	5.4	≈1070	Low cytotoxicity; no obvious organ damage and toxicity	NIR‐II optical/MRI tumor imaging and PDT/PTT tumor treatment	[44]
iRGD‐RM@BPQDs	50 (3 for BPQD)	–	No obvious organ damage and toxicity	PTT tumor treatment	[45]
BP	lateral size ≈2.6, thickness ≈1.5	–	Low cytotoxicity	PTT tumor treatment	[48]
BP	2.5	–	Low cytotoxicity; no obvious organ damage and toxicity	PDT/PTT tumor treatment	[49]
BPQDs@EXO	≈100 (4.0 for BPQD)	–	Low cytotoxicity; no obvious organ damage and toxicity	PTT tumor treatment	[50]
PbS@CdS	–	1500–1700	Low cytotoxicity; no obvious organ damage and toxicity	NIR‐II optical tumor imaging and tumor radiotherapy	[51]
Zn‐doped PbS	2.7‐5.5	1210–1630	Low cytotoxicity	NIR‐II optical vascular imaging	[60]
PbS@CdS	6.9	1600	Cleared through biliary pathway without obvious toxicity	NIR‐II optical vascular imaging	[61]
PbS@CdS	6.8	1600	–	NIR‐II optical vascular imaging and vascular hemodynamics quantification	[62]
PbS@CdS@SiO_2_	≈54 (5 for PbS@CdS)	1155	Low cytotoxicity; no obvious organ damage; slowly degrading in liver to release free Cd and Pb ions.	NIR‐II optical vascular imaging and GI tract imaging	[63]
RNase A@PbS@ZnS	4.9	1600	Low cytotoxicity; cleared through biliary pathway; no obvious organ damage and toxicity	NIR‐II optical vascular imaging	[64]
PbS@CdS	8	1880	–	NIR‐II optical imaging of lymph nodes and venules	[65]
Zn‐doped Ag_2_Te	4.92	1850	Low cytotoxicity; no obvious hemolysis; no obvious organ damage and toxicity	NIR‐II optical vascular imaging and TBI detection	[66c]
Ag_2_Se	3.4	1300	Low cytotoxicity	NIR‐II optical vascular imaging	[67a]
Ag_2_S	5.6	1200	–	NIR‐II optical tumor and vascular imaging	[67b]
Ag_2_Te@Ag_2_S	5.71	1560	–	NIR‐II optical vascular imaging	[68]
ZAISe@ZnS	6.5	530 & 660	Low cytotoxicity; no obvious hemolysis; liver retention	Visible optical atherosclerotic plaque imaging	[75]
Ag_2_S	8	1135	–	NIR‐II optical vascular imaging and ICH detection	[84]
V&A@Ag_2_S	3.3 for Ag_2_S	1050	Low cytotoxicity	NIR‐II optical TBI detection	[87]
Mn^2+^‐doped Ag_2_Te	4.5	≈1690	Low cytotoxicity; no obvious hemolysis; mice body weight decreased slightly after injection and recovered; no obvious organ damage.	NIR‐II optical TBI detection and TBI treatment	[88]
V&C/PbS@Ag_2_Se	≈150 (6.4 for PbS@Ag_2_Se)	1450	Low cytotoxicity; no obvious organ damage and toxicity	NIR‐II optical ischemic stroke detection	[92]
Ag_2_Se QDs‐LnNCs	3.0 for Ag_2_Se QD	1020	Low cytotoxicity; no obvious organ damage and toxicity	NIR‐II optical ischemic stroke and TBI detection	[93]
CdSe@ZnS	–	570 & 615	–	High‐throughput visible optical analysis of viral biomarkers in serum	[101]
CdTe	3.5	610	–	Visible optical detection of EBV capsid antigen IgA in serum	[102]
CdSe@ZnS	–	615	–	Visible optical detection of SARS‐CoV‐2 antibodies level in serum	[104]
CdSe@CdS@ZnS	–	616	–	Visible optical detection of SARS‐CoV‐2 antibodies level in serum	[105]
CdTeSe@ZnS	5.7	750	Low cytotoxicity	NIR‐I optical tracing the viral infections in vivo	[106]
PbS	3	1000	–	NIR‐II optical imaging COVID‐19 infections in vivo	[107]
PbS	–	1300	Low cytotoxicity; no obvious organ damage and toxicity	NIR‐II optical monitoring of ROS/RNS concentrations in viral encephalitis	[108]
ZnCuInSe	–	560–710	Low cytotoxicity; no obvious hemolysis	*Staphylococcus aureus* classification, quantification, and visible optical detection in vitro	[111]
CdSe@ZnS	12	615	–	Visible optical detection of *Streptococcus pneumoniae* in the complex blood sample	[112]
PbS	–	1204–1286	Liver and spleen retention; no obvious organ damage and toxicity	Real‐time NIR‐II optical monitoring of bacterial infection in vivo	[114]
InP	3.216	–	No obvious organ damage and toxicity	Killing bacteria and reducing subcutaneous abscess infection in vivo	[116]
CuInS@ZnS	–	712	Low cytotoxicity; no obvious hemolysis	Bacterial adhesion disruption and biofilm formation inhibition	[117]
CdTe	2.62	–	No obvious organ damage and toxicity	Treatment of the subcutaneous infection	[13a]
CuInSe_2_@ZnS:Al	3.7	750‐981	Low cytotoxicity and minimal foreign body reaction	Invisible NIR‐I medical tattoo for medical data recording	[14]

To date, QD‐based nanomaterials have been widely applied across a number of biomedical areas. The earliest biomedical applications of QDs were mostly focused on cancer diagnosis and therapy since materials with nanometer size were observed to preferentially enter solid tumors via the EPR effect, thereby enhancing interactions with tumor cells while reducing systemic exposure. More recently, QDs have been gradually applied to applications outside of oncology. Owing to their satisfactory hemocompatibility and tunable blood half‐lives, QDs are able to retain in the bloodstream for a long time and are sensitive to vascular micro‐disruption, enabling the detection of diseases characterized by vascular dysfunction. Through optical imaging—especially in the NIR‐II imaging window—the fine anatomical structure of the vasculature has been successfully delineated. This strategy has also been used in other vascular disease applications, such as the in vivo diagnosis of atherosclerotic plaques, thrombolysis, stroke, cerebral hemorrhage, and traumatic brain injury in rodent models. In recent years, due to the continually improving understanding of disease pathology at the molecular level, as well as improvements in synthesis, regulation, and characterization technology, the biomedical applications of QDs have been further broadened to address clinical challenges such as the rapid biosensing of bacterial and viral infections, monitoring of disease progression, and encoding readily accessible and reliable medical history.

Nevertheless, although preclinical studies with QD‐based nanomaterials have shown exciting potential, translating these technologies to the clinic has been very challenging. Biocompatibility and toxicity have been expressed as potential concerns for QDs given their nanoscale size. In brief, the toxicity of QDs is primarily a consequence of the leaching of toxic heavy metal ions. A number of studies have shown that surface modification can reduce QD toxicity by constructing core@shell or core@shell@shell structures by encapsulating the heavy metals with low‐toxicity or nontoxic inorganic or organic polymers or by altering surface chemistry. Additionally, a variety of biocompatible heavy metal‐free QDs, such as I–VI and I–III–VI compounds, mostly based on elements, such as Cu, Ag, In, Zn, P, S, and Se, have been explored. In general, these heavy metal‐free QDs have demonstrated substantially improved safety profiles compared to heavy metal‐containing QDs. The reduced toxicity of heavy metal‐free QDs has also been discussed previously,^[^ ^10^ ^]^ and more information on heavy metal‐free QDs can be found in a recent review paper.^[^ ^5^ ^]^ According to previous studies, after administration into the body (especially through the intravenous route), a large fraction of nanomaterials remain trapped within the reticuloendothelial system, leading to potential toxicity.^[^ ^128^ ^]^ Previous studies have shown that small‐diameter nanomaterials can be cleared by the kidney.^[^ ^129^ ^]^ Fortunately, QD‐based probes can be designed with very small diameters (usually < 10 nm), especially when QDs are composed of semiconductor materials with small exciton Bohr radii. As a result, it may be possible to achieve safe and time‐limited QD theranostics without undesired retention in the liver, spleen, and lungs.^[^ ^130^ ^]^ Therefore, the multiple factors including particle size, renal clearance rate, and practical performance must be properly balanced to not only meet the technical needs of a particular application but also ensure a favorable safety profile. Apart from safety concerns, the large‐scale production of QD probes with high reproducibility remains difficult because QD synthesis procedures usually involve a series of chemical and physical processes, which are affected by many parameters, such as temperature, time, heat transduction, and mass transduction. Finally, as with all preclinical in vivo studies, disease models in rodents may yield very different data than those performed in humans. Therefore, QD‐based nanoprobes should be further assessed on large animal models, such as macaques, dogs, and/or swine, especially for evaluating whether their diagnostic and therapeutic capabilities are still valid. Even so, there is still ample reason to believe that QD‐based technologies will become useful tools in the clinic, providing better options for clinicians and patients in the future.

## Conflict of Interest

The authors declare no conflict of interest.
